# Impacts of environmentally relevant concentrations of antibiotic cocktails on the skin microbiome of Eurasian carp (*Cyprinus carpio*)

**DOI:** 10.1186/s42523-025-00434-8

**Published:** 2025-07-08

**Authors:** Ashley G. Bell, Emma R. Vaughan, Barbara Kasprzyk-Hordern, Jo Cable, Ben Temperton, Charles R. Tyler

**Affiliations:** 1 Biosciences, Faculty of Health and Life Sciences, University of Exeter, Exeter, Devon, EX4 4QD UK; 2 Sustainable Aquaculture Futures, University of Exeter, Exeter, Devon, EX4 4QD UK; 3 Department of Chemistry, University of Bath, Claverton Down, Bath, BA2 7AY UK; 4 School of Biosciences, Cardiff University, Cardiff, CF10 3AX UK

## Abstract

**Background:**

The skin surfaces of fish harbour diverse assemblages of microbes (microbiomes) that play critical roles in host health and disruption of these microbiomes can lead to disease conditions. Antibiotics, widely used in medicine for human and animal health treatments, are increasingly found in waterways and this is a growing concern due to their potential to alter the balance of microbial ecosystems and drive antimicrobial resistance (AMR). The effects of antibiotics on skin microbiomes in fish, however, have been little explored. This study examines how exposure to environmental levels of antibiotics affects the skin microbiomes of Eurasian carp (*Cyprinus carpio*).

**Results:**

A 2-week exposure of Eurasian carp to cocktails of five antibiotics (ciprofloxacin, clarithromycin, sulfamethoxazole, trimethoprim, and tetracycline) at concentrations found in the environment resulted in significant skin bacterial community compositional shifts. Applying 16S rRNA amplicon sequencing, we found enrichment of the genus *Arcicella* (Proteobacteria) and depletion of *Sphaerotilus* (Bacteroidetes) with limited recovery even after maintaining the fish for a further two weeks in clean (antibiotic-free) water. In the low-antibiotic concentration exposure group, the tank water microbiome assemblages resembled those of the fish skin suggesting similar responses to the antibiotic treatments. Metagenomic analysis observed no increase in antibiotic resistance genes or changes in metabolic pathway abundance, possibly due to the relatively short duration of antibiotic exposure.

**Conclusion:**

This study highlights that even low-level exposure to chemical mixtures can alter fish skin microbiome compositions, with limited recovery observed after cessation of exposure. These findings warrant further assessments of the long-term effects and functional consequences of these altered microbiomes on fish health, particularly in environments increasingly affected by anthropogenic chemical pollution.

**Supplementary Information:**

The online version contains supplementary material available at 10.1186/s42523-025-00434-8.

## Introduction

Antibiotics include a wide range of naturally and synthetically produced chemicals that inhibit (bacteriostatic) or kill (bactericidal) bacteria. They are classified as narrow-ranging (such as clarithromycin and erythromycin) or broad-ranging (such as amoxicillin and ciprofloxacin) based on the microbes they target. Antibiotics are used widely in farming and human healthcare and their usage is growing; global human antibiotic consumption grew by 65% to 34.8 billion defined daily doses between 2000 and 2015 [46]. Administered antibiotics are often not fully metabolised and a significant proportion is excreted into our sewerage system as the parent compound or its metabolites [48, 49]. This is compounded globally as more than 45% of human waste (in 2020) received no treatment before being discharged into the environment [104]. Even where wastewater treatment processes are in operation, events such as storms can overwhelm wastewater treatment resulting in untreated effluent entering waterways from combined sewer overflows [14, 38, 73]. Illustrating this in 2023 in the UK, sewage outflows resulted in 464,056 discharge events for a combined 3,606,170 h of discharge, a rise of 54% and 105% respectively compared to 2022 (Environment [30]). As a consequence of these activities, antibiotics are widely and increasingly found in surface waters globally [2, 6, 40, 82, 85, 98]. Furthermore, by 2030, it is estimated that the use of antibiotics in agriculture and aquaculture will be over 121,000 tonnes globally (aquaculture constituting around 9% of this, at 13,600 tonnes [66, 88],). In aquaculture, antibiotics are often administered inappropriately through feed or water and at high (therapeutic) levels prophylactically and in response to infection risk or disease [27, 36, 41, 88].

Microorganisms in surface waters, including those that are free living in the water column, in biofilms and in the microbiomes of metazoans play vital roles in ecosystem function. In metazoans, such as fish, assemblages of microorgansims (microbiomes) are critical for health providing protection from disease and priming the immune system [9, 43, 52]. Microbiomes, however, are highly susceptible to the effects of broad-spectrum antibiotics that are indiscriminate, killing both commensal and pathogenic taxa alike [18, 23, 24, 53, 54, 83, 84]. The effects of antibiotics on fish gut microbiomes have been studied, especially in aquaculture, but less is known about the effects of environmentally relevant concentrations of antibiotics on fish skin microbiomes. Furthermore, the few studies addressing this have been conducted using therapeutic dosages several orders of magnitudes higher than those recorded in the environment [18, 23, 24, 61].

Although environmental concentrations of antibiotics tend generally to be at low levels (~ 100 ng/L, see Supplementary Table 1; [3, 4, 10, 51, 95, 97]), they can nevertheless select for antimicrobial resistance (AMR) at sub-minimum inhibitory concentrations for bacterial growth [34, 45, 67, 68, 93]. Additionally, antibiotics in waterways are almost always present in combination and can have additive effects, increasing selection pressure and AMR [15, 62].

The mucosal surface of fish hosts a complex microbial community, termed the skin microbiome, whose composition is mediated by both host-specific (such as host genetics) [17, 86] and environmental conditions (such as freshwater vs marine) [47, 56, 63]. While variable, fish skin microbiomes are typically dominated by bacterial phyla such as Proteobacteria, Actinobacteria, Bacteroidetes and Firmicutes [32, 53, 54]. Functionally, this community plays important roles in nutrient cycling, modulating the host’s immune system and colonisation resistance against opportunistic pathogens [16, 43, 65]. Understanding this complex host-microbe system and its drivers is essential in evaluating the impact of stressors such as exposure to environmental contaminants like antibiotics.

Studies to date on the effects of antibiotics on fish skin microbiomes have focused on rifamycin and tetracyclines, antibiotics not widely found within the environment. Macrolides, quinolones, sulphonamides and trimethoprim antibiotics are some of the most commonly detected antibiotic families in surface waters [10, 13, 28, 87, 100] with macrolides and fluoroquinolones found at the highest average concentrations [5, 39, 92, 101], but these are far less studied. Fish microbiome studies have generally focused on aquaculture species including the seabass (*Dicentrarchus labrax*) [83, 84], Nile tilapia (*Oreochromis niloticus*) [61] and yellowtail kingfish (*Seriola lalandi*) [53], or model organisms such as the mosquitofish (*Gambusia affinis*) [23, 24]. Different species of fish have been shown to have different skin microbial compositions, a term called phylosymbiosis [17], and therefore, extrapolations between species are largely restricted to broad effect analyses, rather than to individual taxa.

While the detrimental effects of antibiotics on gut microbiomes are well-studied, less is known about their impact on the fish skin microbiome. To address this gap, we investigated how environmentally relevant antibiotic mixtures affect the skin microbiome of the globally important aquaculture species, Eurasian/common carp (Cyprinus carpio). Carp are found in surface freshwaters across wide global areas and are also the most farmed fish globally by tonnage and value, especially in low- and middle-income countries [31]. Our central hypotheses were that chronic exposure to environmentally relevant antibiotic cocktails representing common classes (clarithromycin (macrolide), ciprofloxacin (fluoroquinolones), sulphamethoxazole (sulphonamides), trimethoprim and tetracycline) would: (i) result in clear shifts in the skin microbial community structure, (ii) increase the abundance of corresponding antimicrobial resistance genes (ARGs); and (iii) the skin microbial community structure would persist and not return to the pre-exposure state following a depuration period. We tested these predictions by exposing carp to two distinct, environmentally relevant antibiotic mixture concentrations under flow-through conditions for two weeks, followed by a two-week recovery period. Longitudinal changes were monitored by analysing skin swabs using 16S rRNA amplicon sequencing for community composition and shotgun metagenomics to assess ARG loads and metabolic profiles.

## Methods

The full methods can be found in supplementary documents.

### Ethics

All experiments were performed with the approval of the University of Exeter Research Ethics and Governance Review Board (Application ID 1549617) and conformed to UK legislation under the Animals (Scientific Procedures) Act 1986 (ASPA).

### Fish source and maintenance

A total of 72 Eurasian carp (*Cyprinus carpio*) with a mean total fork length of 13.4 cm (SD ± 0.82 cm) and a mean weight of 61.6 g (SD ± 11.45 g) were housed temporarily for four weeks in 250 L opaque circular tanks at the University of Exeter Aquatic Research Centre. Carp were fed once daily at 16:00 with a commercial sinking 4 mm shrimp-based pellet (The Aqua Shack) at 2% fish bodyweight per day. Tanks were supplied with dechlorinated mains tap water, which was aerated and maintained at 13 °C using a flow-through system. Dechlorinated freshwater was monitored for pH, temperature, conductivity, dissolved oxygen, ammonia, nitrate, nitrite and alkalinity weekly to ensure they were within guidelines set by the U.S. EPA [96]. The photoperiod was set to 12 h:12 h light:dark 0800:2000, with an artificial dawn/dusk transition of 30 min.

### Experimental setup

**Fig. 1:**
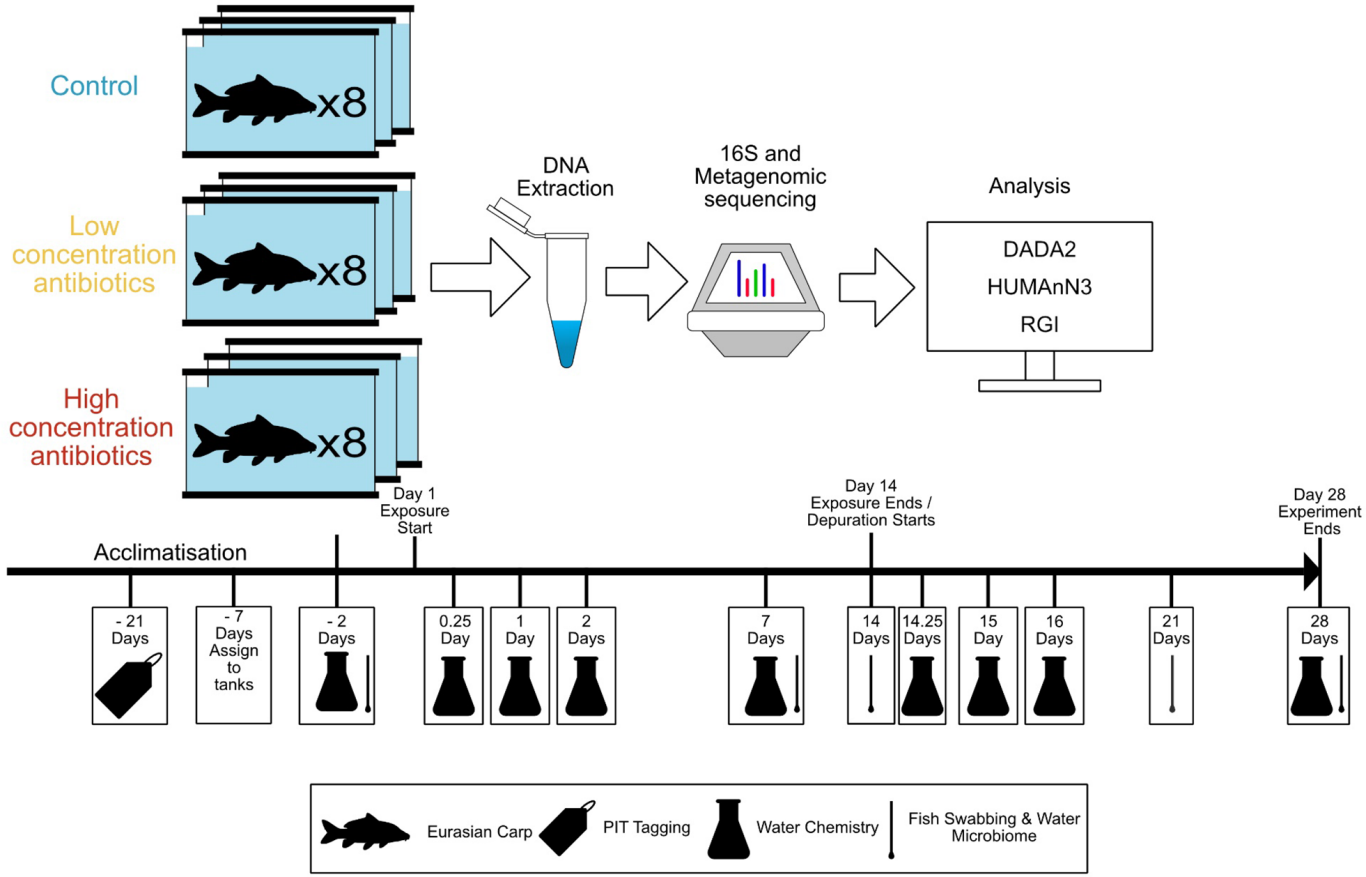
Overall experimental design schematic. Seventy-two Eurasian carp were PIT tagged three weeks before the antibiotic exposure. One week before initiating the exposure, carp were randomly assigned to experimental tanks. Carp were then exposed to two antibiotic cocktails at two different dosages and a control. Carp were exposed for a total of two weeks followed by two weeks of depuration in antibiotic-free water. Fish skin swabs and water microbiome samplings were taken at − 2, 7, 14, 21 and 28 days after initiating the antibiotic exposure. Water samples for determining antibiotic concentrations are taken at − 2, 0.25, 1, 2, 7, 14.25, 15, 16 and 28 days after initiating the antibiotic exposure. Created in BioRender

The antibiotic exposure experiment was conducted between June and July 2023. After four weeks of acclimation to the temporary housing tanks, carp were anesthetised using 70 mg/L benzocaine, weighed and measured for body length, and individually tagged with a BioMark N125 Passive Integrated Transponders (PIT) Tag system which consisted of a 1 cm radio-frequency identification (RFID). The tag was implanted in the musculature below the surface of the fish skin behind the dorsal fin. After a two-week recovery period and one week before the experiment start date, carp were randomly transferred to one of the nine 100 L rectangular experimental glass tanks which comprised three treatment groups (with three replicate tanks for each treatment), consisting of a low-concentration antibiotic cocktail, a high-concentration antibiotic cocktail and a freshwater control (Table 1). The low concentration antibiotic cocktail (targeted for 1.25 μg/L of total antibiotics) consisted of 0.75 μg/L ciprofloxacin (Cayman chemical; CAY14286), 0.2 μg/L clarithromycin (Cayman chemical; CAY19455), 0.1 μg/L sulfamethoxazole (LKT Labs; s8248), 0.1 μg/L trimethoprim (Cayman chemical; CAY16473), and 0.1 μg/L tetracycline (LKT Labs; t1679). The high concentration cocktail (targeted for 6.25 μg/L of total antibiotics) consisted of 3.75 μg/L ciprofloxacin, 1 μg/L, clarithromycin, 0.5 μg/L, sulfamethoxazole, 0.5 μg/L trimethoprim, and 0.5 μg/L tetracycline. Eight carp were placed into each of the three replicate treatment tanks (Fig. 1). These antibiotic cocktails represent the median concentration for measured antibiotic concentrations recorded globally within wastewater treatment plant (WWTP) effluent retrieved from the literature for studies between 2010 and 2019 (Supplementary Table 1), and one at five times the median concentration to provide levels relevant to an untreated WWTP effluent.

**Table 1:** Mean antibiotic exposure concentrations during the exposure period

Exposure group	Antibiotic	Mean exposure concentration (µg/L)	Standard deviation (µg/L)
High	Ciprofloxacin	8.96	± 4.46
High	Clarithromycin	1.79	± 0.98
High	Sulphamethoxazole	0.67	± 0.36
High	Tetracycline	1.04	± 1.12
High	Trimethoprim	0.72	± 0.43
Low	Ciprofloxacin	2.02	± 0.94
Low	Clarithromycin	0.36	± 0.17
Low	Sulphamethoxazole	0.12	± 0.06
Low	Tetracycline	0.15	± 0.09
Low	Trimethoprim	0.18	± 0.08
Negative control	Ciprofloxacin	0.00	± 0.00
Negative control	Clarithromycin	0.00	± 0.00
Negative control	Sulphamethoxazole	0.00	± 0.00
Negative control	Tetracycline	0.00	± 0.00
Negative control	Trimethoprim	0.00	± 0.00

### Antibiotic exposure

Low and high concentration antibiotic cocktails exposure stock solutions were prepared at 3600 × final exposure concentrations in ultrapure water (Elga PURELAB Flex 3 system). All antibiotic exposure stocks were made up in one batch and aliquoted at the beginning of the experiment (to ensure dosing consistency) and frozen at − 20 °C. All antibiotic dosing stocks were made up and replaced every two days by adding ultrapure water to frozen antibiotic exposure stocks in 2 L Duran bottles covered in aluminium foil to reduce UV degradation. To administer antibiotics into exposure tanks, dechlorinated freshwater was fed from a header tank system into an aspirator (acting as a mixing cell) at a 3600:2 ratio of dechlorinated freshwater to antibiotic cocktail, respectively (Supplementary Fig. 1). Antibiotics from stock solutions were administered to the aspirator using a peristaltic pump (Watson Marlow 530 series, 520R pump head) using PTFE and Marprene tubing (Watson Marlow) to reduce antibiotic adsorption. Aspirators contained a PTFE magnetic flea set to 120 rpm to ensure adequate mixing. A splitter tank then divided the aspirator outflows through glass tubing into experimental tanks. Water flow rates for each tank were set to six tank changes daily (600 L/24 h).

### Biological sampling

Carp skin microbiome samples were taken periodically from individually PIT-tagged carp (Supplementary Fig. 2). These samples were taken 2 days before the antibiotic exposures commenced and at 7- and 14-days of the exposure. After 14 days, antibiotic exposure was ceased, and further skin swab samples were taken at 7- and 14- days after the exposure when the fish were held in clean (antibiotic free) water (depuration; 21 and 28 days since the start of exposure; see Fig. 1). This resulted in a total of five sampling time points (1 pre-exposure, 2 during antibiotic exposure and 2 during depuration). For the collection of skin swabs the carp were removed from the tank, the RFID tag recorded, and swabbing carried out on the right side of the fish. The swabbing consisted of swabbing with a sterile cotton swab (TS/8-A; Technical Service Consultants Ltd) running from just below the top fin to the caudal fin following the direction of the scales, rotating the swab. This was repeated five times each time taking a different line across the body of the carp to ensure the same location was not swabbed twice. The process took less than 10 s. Swab tips were snapped off using sterile techniques and stored in sterile 2 mL Eppendorf tubes containing 570 μL of lysis buffer (30 mM Tris–HCl, 30 mM EDTA, pH 8) and 0.5 g garnet beads. Eppendorfs were immediately snap-frozen in liquid nitrogen and subsequently stored at − 80 °C until processing for DNA extraction. After sampling carp were transferred to a clean holding tank with clean dechlorinated water before their return to the original treatment tanks after all fish had been sampled. To assess tank water microbial content, at all fish sampling time points, a total of 1 L of tank water was collected in duplicate (total 2 L) and passed through a filter (47 mm diameter, 0.4 μm pore size, 7060–4704, Whatman) using a 50 mL syringe. Filters were immediately stored in 2 mL Eppendorf tubes with 570 μL of lysis buffer and 0.5 g garnet beads. Eppendorf tubes were immediately frozen in liquid nitrogen and subsequently stored at − 80 °C until processing for DNA extraction.

### Antibiotics analysis of the water

Water samples were taken to confirm antibiotic dosing concentration in the exposure tanks. Water samples were taken 2 days before the antibiotic exposure was started, after 6, 12 and 24 h, and then 7 days into the exposure, and at 6, 12 and 24 h and 14 days (Day 28) during the depuration phase. These time points were selected to ensure no antibiotics were present in the system before the experimental start and to see how quickly antibiotics reached our target concentration and subsequently cleared from the test system upon cessation of dosing. All experimental equipment was rinsed in ultrapure water, methanol then ultrapure water again to remove organic residues.

### DNA extraction and 16S rRNA sequencing

DNA was extracted from carp skin swabs, tank water microbes captured on filters, positive (ZymoBIOMICS Microbial Community Standard (Zymo; D6300) and negative (blank swabs/filter) controls using an in-house CTAB/EDTA/chloroform method (adapted from Chaput [26] and Lever et al., [55]. The full protocol is available at https://dx.doi.org/10.17504/protocols.io.bw8gphtw.

The V4 region (515F–806R) of 16S rRNA was amplified along with a positive, negative (filter and swab) and no template control (NTC) control for each 96-well plate. 96 primer pairs for the 16S V4 region pairs (515F–806R) were constructed (from Integrated DNA Technologies) ensuring each well within a plate was dual indexed to reduce index hopping (carried out using the updated Earth Microbiome Project primers; [21, 22, 74, 79, 99]) (Supplementary Table 1). After 16S V4 amplification, an Illumina barcode was ligated to equimolar pooled amplicons from the same plate to allow for multiple plates with the same indexes to be used, as applied in Guenay-Greunke et al. [33]. All PCRs were performed in duplicate. After amplification, duplicate PCR reactions were combined and 16S V4 regions were checked for size (~ 319 bps) to ensure there were no spurious bands, using standard gel electrophoresis methods. Failed PCRs were repeated. PCR products were cleaned using a magnetic bead clean-up protocol as detailed in J. McMurtrie et al. [60] and pooled to 50 ng/µL in equimolar amounts. Pooled plates were submitted to the University of Exeter Sequencing Service for Illumina barcoding and sequencing. Amplicon barcoding consisted of PCR-free ligation of Illumina adaptors (plate barcoding) and amplicon sequencing on the NovaSeq SP 2 × 250 paired ends (500 cycles). 50,000 reads per sample was targeted for all amplicons from fish skin swab samples. The data for this study have been deposited in the European Nucleotide Archive (ENA) at EMBL-EBI under accession number PRJEB82662.

### 16S Demultiplexing and bioinformatic processing

All bioinformatic processes and subsequent analyses can be found online at: https://github.com/ash-bell/fish_skin_antibiotic_exposure and in the supplementary documents. Shotgun metagenomic sequencing was performed separately from the amplicon sequencing. This consisted of a total of 26 samples including twelve fish skin swabs, twelve tank water samples and two negative controls from the Day 14 sampling timepoint. This was comprised of four samples from the control tanks, the low-concentration exposure and the high-concentration exposure tanks. Samples were sent for library preparation and sequencing at the Centre for Genomics Research, Birmingham, UK. All samples were sequenced on three separate Illumina NovaSeq runs using SP chemistry (Paired-end, 2 × 150 bp sequencing). Library preparation of gDNA consisted of the NEBNext Ultra II FS Kit (1/2 volume reactions) with ~ 350 bp inserts. An average of 1,146,982,690 trimmed reads per swab sample (standard deviation: ± 148,994,417) and 277,880,774 reads per filter (standard deviation: ± 51,075,475) were obtained.

### Bioinformatic analysis

All statistical analyses were performed in R v4.3.2 [80] with data manipulation performed using tidyverse v2.0.0 [102]. Figures were constructed using ggplot2 v3.4.4 (Hadley [35]), microViz 0.11.0 [7], and patchwork v1.2.0 [77]. All linear mixed model statistical tests were performed using lmerTest v3.1–3 [50] with the interaction of treatment and days post exposure start as the fixed effect and a random slope and effect of treatment with treatment nested within tank and PIT tag number (except for filters) (“~ treatment * Days.post.exposure.start + (1+treatment | treatment/tank/PIT.tag.num”). The normality of linear model residuals was confirmed using a Q-Q plot and heteroskedasticity plotting the least squares residual against the independent variable. All statistical tests with multiple re-testing were corrected for false discovery rate using the Benjamini & Hochberg [11] method [11]. Amplicon data were manipulated using the phyloseq v1.46.0 [59] and microViz. Phylogenetic trees were rooted using the longest branch using the ape v5.7–1 [75] to ensure reproducibility. Positive controls (ZymoBIOMICS Microbial Community DNA Standard) were compared against manufacturer’s expected microbial community composition to ensure even DNA extraction (Supplementary Fig. 3). Decontam v1.22.0 [29] was used to remove presumed contaminating ASVs identified either through the frequency or prevalence method at 0.05 and 0.25 thresholds respectively. 16S ASVs identified at a family level as Mitochondria, order level as Chloroplast or domain level as Eukaryota were discarded.

ASV tables were rarified through a rarefaction method which resampled without replacement each ASV Table 1000 times using 95% of the lowest sample size (9491 × 0.95 = 9016 ASV) [89, 90]. This ensured each sample was sampled evenly such that normalisation was not required. All metrics (alpha and beta diversity, differential abundance) were calculated on each of the 1000 rarified ASV tables and the mean of all alpha or beta diversity replicates was used. Alpha diversity was calculated on each of the raw rarified ASV tables with no transformation using Shannon and Chao1 diversity measured using phyloseq and Faith’s phylogenetic diversity using the picante v1.8.2 [44]. Beta-diversity was also calculated on each of the 1000 raw rarified ASV tables with no transformation using microViz for either a Bray–Curtis (vegan v2.6.4) [72], Weighted UniFrac (GUniFrac v1.8) [57, 103], Jaccard or Aitchison dissimilarity matrix aggregated at a genus level, (except for any UniFrac measurement which requires no aggregation). Beta-diversity statistics were calculated using a PERMANOVA using the adonis2 function from the vegan with post-hoc style test performed using a pairwise.adonis2 v0.4.0 wrapper script [78]. Beta-dispersions of treatment groups calculated from a Bray–Curtis dissimilarity matrix were determined using microViz. Differential abundance of taxa was calculated on each of the raw 1000 ASV tables counts with no transformation using a linear mixed model with random effects and corrected for false discovery rates as described above using microViz.

Shotgun metagenomics was first processed using bbmap v39.01 bioinformatics suite [19]. Common sequencing adaptors, known sequencing artifacts and PhiX spike-ins sequences were first removed from samples bioinformatically using bbduk.sh. BBmap.sh was then used to bioinformatically remove carp (NCBI database: GCA_018340385.1) and human (NCBI database: HG19) genomic data masked for homologous bacterial sequences [20]. Samples were then error-corrected using tadpole.sh. Samples from different sequencing runs were then concatenated and assembled together using metaspades.py v3.15.5 [71]. Genes from subsequent assemblies were determined using prodigal v2.6.3 [37] in metagenomics mode. ARGs were determined from gene protein translations using the resistance gene identifier (RGI) v6.0.2 [1]. Metagenomic profiles from error-corrected reads were determined using HUMAnN3 [8] following their standard protocol with metabolic profiles from MetaCyc [25]. Metabolic profiles for each sample were compared using a Bray–Curtis dissimilarity matrix and differential abundance using a linear mixed model with the formula “lmer(CPM~treatment.group+(1|tank)”. Models were corrected for false discovery rate using the Benjamini & Hochberg method with a *p*-value cutoff of 0.05.

## Results

### Water chemistry

Antibiotic concentrations in exposure tanks were higher than anticipated throughout the experiment, but still within the regime of environmentally relevant levels (Table 1). The measured concentrations averaged 1.85 (SD ± 0.675) and 2.22 (SD ± 0.219) times above the nominal levels for the high and low concentration exposure respectively during the exposure period (Supplementary Fig. 4). Target concentrations were reached within six hours of initiating the exposure and antibiotics had been cleared from the tank water between 6 and 24 h during the depuration period. Physiochemical water parameters remained relatively stable throughout the experiment (alkalinity mean 3.64 dKH ± 0.55, ammonia mean 0.04 mg/L ± 0.03, temperature mean 12.16 °C ± 0.19, conductivity mean 292.6 µS/cm ± 10.92, dissolved oxygen mean 6.53 mg/L ± 0.54 or mean 61.67% ± 4.64, nitrate mean 7.02 ppm ± 6.39, nitrite mean 0.01 ppm ± 0.02, pH 7.49 ± 0.18. (± as standard deviation)). No significant differences in carp growth rate (length and weight) were observed between the control and treatment groups at the end of the experiment. Average daily body growth across treatments was: control (0.37 mm, 0.59 g), low concentration (0.32 mm, 0.54 g), and high concentration (0.35 mm, 0.56 g) for fork length and weight, respectively.

### Alpha diversity is not impacted by environmentally relevant concentrations of antibiotics

**Fig. 2:**
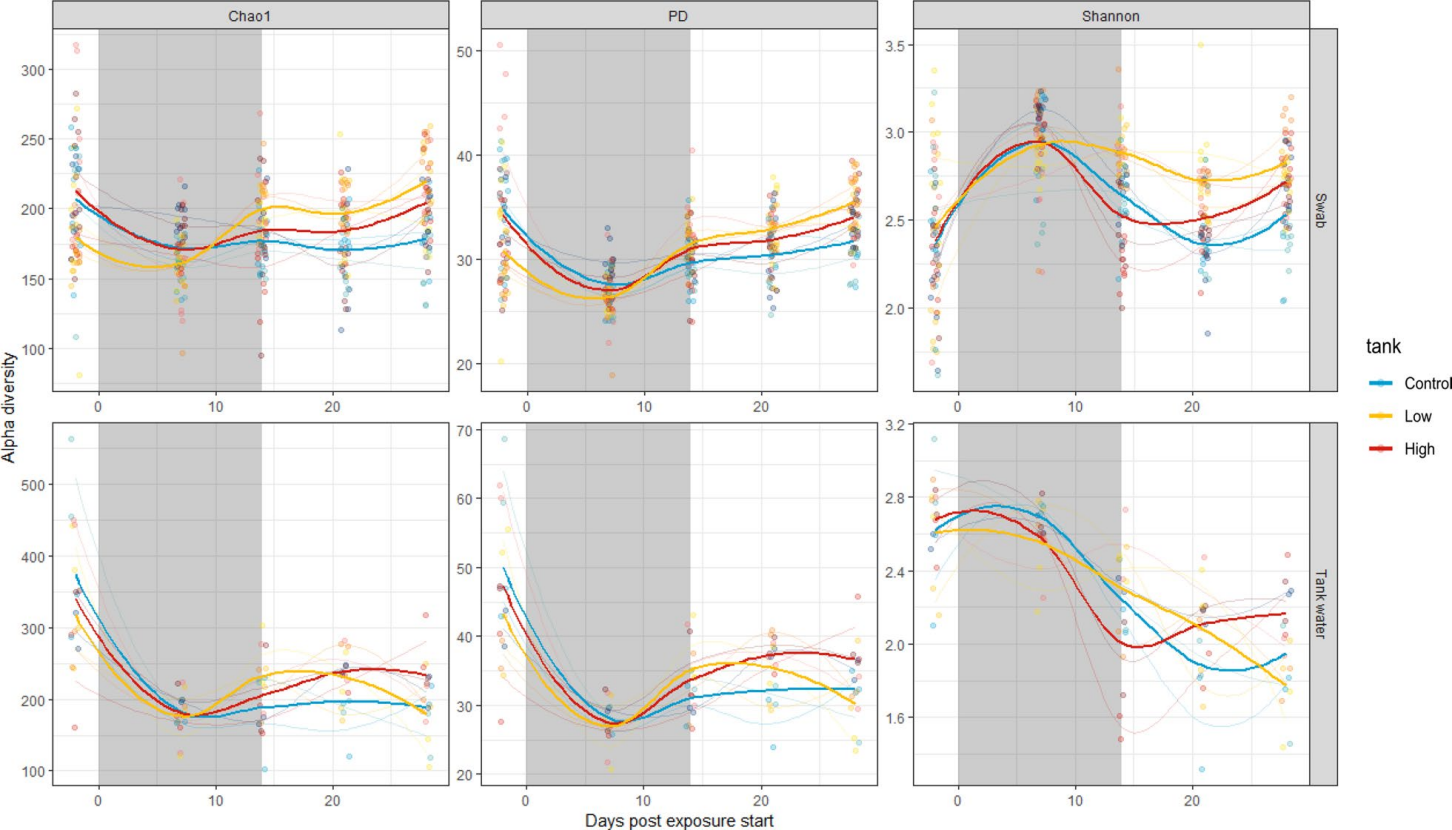
Alpha Diversity of Carp Skin and Tank Water Microbiomes. Carp skin swabs are in the upper half of the figure and tank water microbiomes the bottom half. Alpha diversity (Chao1, Shannon index, and Faith’s phylogenetic distance (PD)) of carp skin and tank water bacterial communities over a two-week antibiotic exposure period followed by a two-week depuration period. The grey shaded area indicates the duration of antibiotic exposure. Trend lines represent the locally estimated scatterplot smoothing (LOESS) regressions for each treatment group

Three alpha diversity metrics were evaluated to assess the impact of antibiotic exposure on the microbiome community richness and evenness in both carp skin and tank water microbiomes: Chao1, Faith’s phylogenetic diversity (PD), and Shannon index. No significant differences in any of these alpha diversity metrics were observed between treatment and control groups at any time point (Fig. 2, Supplementary Table 3). These findings suggest that environmentally relevant antibiotic concentrations do not alter alpha diversity in carp skin or tank water microbiomes.

### Antibiotics alter the microbial community composition (beta diversity) of the carp skin microbiome and are more varied following depuration

**Fig. 3:**
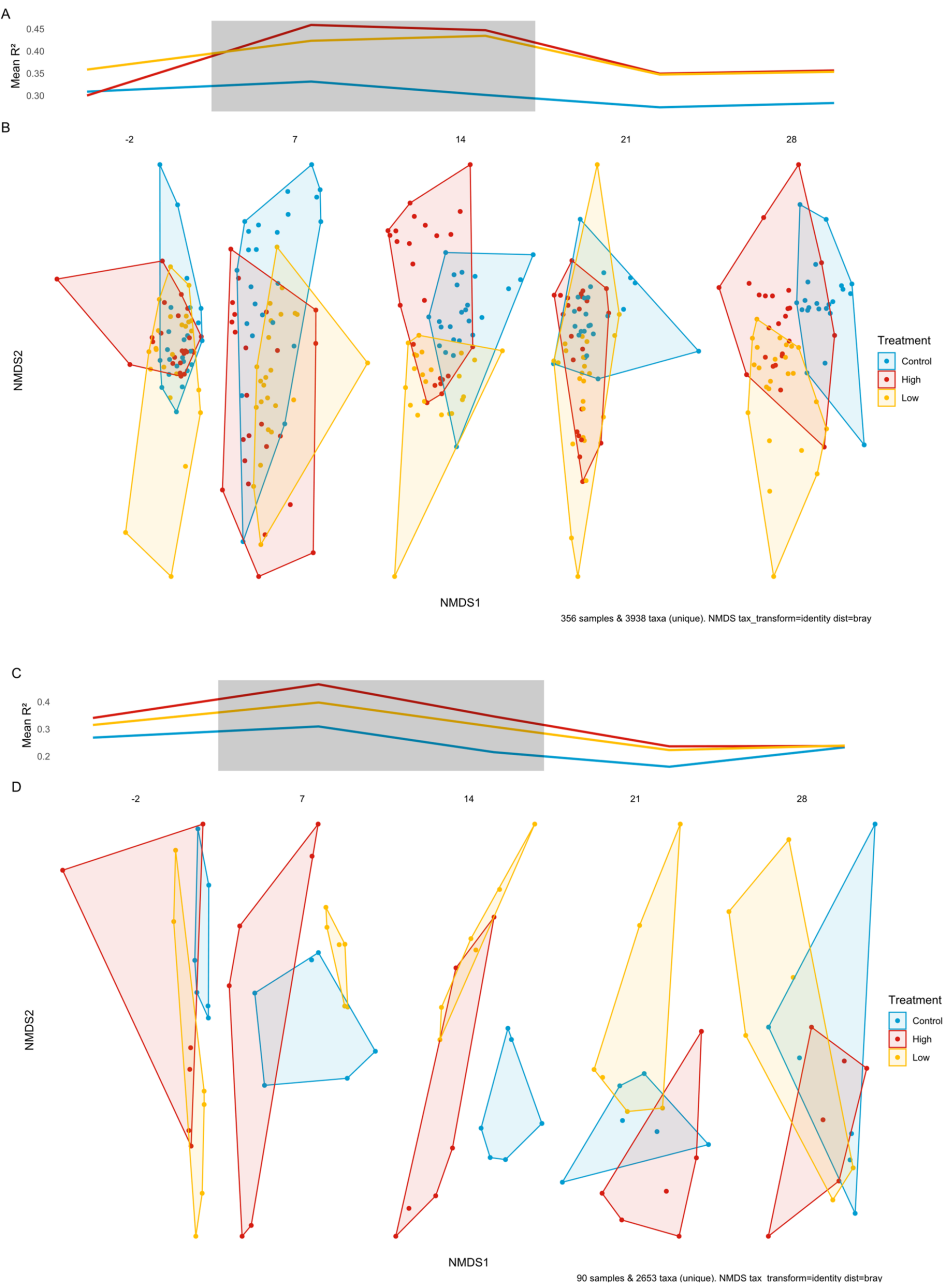
Impact of antibiotic exposure on microbiome beta diversity. The grey box indicates the period of antibiotic exposure. A&C) Average difference (Bray–Curtis dissimilarity) between control (blue), low-concentration (yellow), and high-concentration (red) antibiotic exposure groups compared to the control group over time. Data is based on 1000 rarefied ASV tables using raw ASV counts. The higher the dissimilarity value, the greater the difference in microbial community composition between the treatment groups and the control. **A** Fish skin swabs; **C** Tank water. **B** and **D** Non-Metric Multidimensional Scaling (NMDS) visualising the overall differences in microbial community composition between control (blue), low-concentration (yellow), and high-concentration (red) antibiotic exposure groups of microbial communities. Each data point represents a sample, and closer points indicate more similar communities. **B** Carp skin swabs; **D** Tank water microbiomes (filters)

Beta diversity was assessed in carp skin and tank water microbiomes to determine the impact of antibiotic exposure on bacterial community composition (Fig. 3, Supplementary Table 4, Supplementary Table 5). Carp skin microbiomes were the most dissimilar between the antibiotic treatment groups and the control. After seven days of antibiotic exposure, there was a greater dissimilarity in the beta diversity between the controls and antibiotic treatment groups compared to the pre-exposure timepoint (R^2^ = 26.76%, *p* = 0.003; R^2^ = 27.68%, *p* = 0.003 low and high concentration respectively). This dissimilarity was even greater after 14 days of antibiotic exposure, in the high concentration exposure versus control (R^2^ = 33.39%, p = 0.003) indicating further divergence in the skin microbiome for this exposure.

Following seven days of depuration (Day 21), there was a decrease in carp skin microbiome dissimilarity metric (R^2^) between the control and treatment groups; the R^2^ value for the control versus low concentration exposure was 12.19% (*p* = 0.003) and for the control versus high concentration exposure 12.97% (*p* = 0.003). However, even after two weeks of depuration (Day 28) the beta diversity composition had a higher degree of difference to that of the pre-exposure period. The dissimilarity between the control and low concentration (R^2^ = 18.27%, *p* = 0.003) and high concentration (R^2^ = 20.15%, *p* = 0.003) exposure groups were more dissimilar to the control group than in the pre-treatment time point.

### Bacterial community composition (beta diversity) of the tank water microbiome

The beta diversity of the microbiome in the tank water did not differ between treatment and control groups at the outset of the experiment prior to the antibiotic dosing period (Fig. 3, Table 1). After one week of exposure, the low antibiotic concentration treatment groups displayed a non-significant but large divergence in beta diversity compared to the control (R^2^ = 19.59%, *p* > 0.05, not significant). After two weeks of exposure, there was a significant increase in the microbiome dissimilarity between low-concentration exposure and control, whilst in the high-concentration exposure the level of dissimilarity did not differ from that at the one-week time point (R^2^ = 42.47%, *p* = 0.006, R^2^ = 39.66%, *p* = 0.006 respectively). After one week of depuration, water microbiomes in both low and high-concentration antibiotic treatment groups exhibited similar beta diversity to those in the pre-exposure and with no differences from the control group (R^2^ = 12.12% and R^2^ = 15.58% respectively, both *p* > 0.05, not significant). This was also the case after 14 days of depuration (control versus low concentration exposure R^2^ = 14.83%; control versus high concentration exposure R^2^ = 17.30%; both *p* = not significant, *p* > 0.05).

The beta diversity analyses were repeated using different distance metrics sensitive to different aspects of beta diversity. These metrics included Weighted UniFrac (incorporating phylogenetic relationships), Aitchison’s distance (accounting for log-scale changes in abundance), and Binary Jaccard (only presence/absence of taxa). These analyses yielded similar results to those obtained with the Bray–Curtis dissimilarity metric used previously (Supplementary Fig. 7).

### Differential abundance of microbial taxa under antibiotic exposure characterised by *Sphaerotilus* and *Arcicella* genera abundance

Differential abundance analysis was performed for microbial taxa at each time point compared with the control group to determine which taxa were associated with the changes in beta diversity under antibiotic exposure and during depuration. Here, only genus and phylum levels are displayed on differential abundance trees, with the full data available in Supplementary Table 4. Genera with clear patterns are described here and further details can be seen in the supplementary documents.

**Fig. 4:**
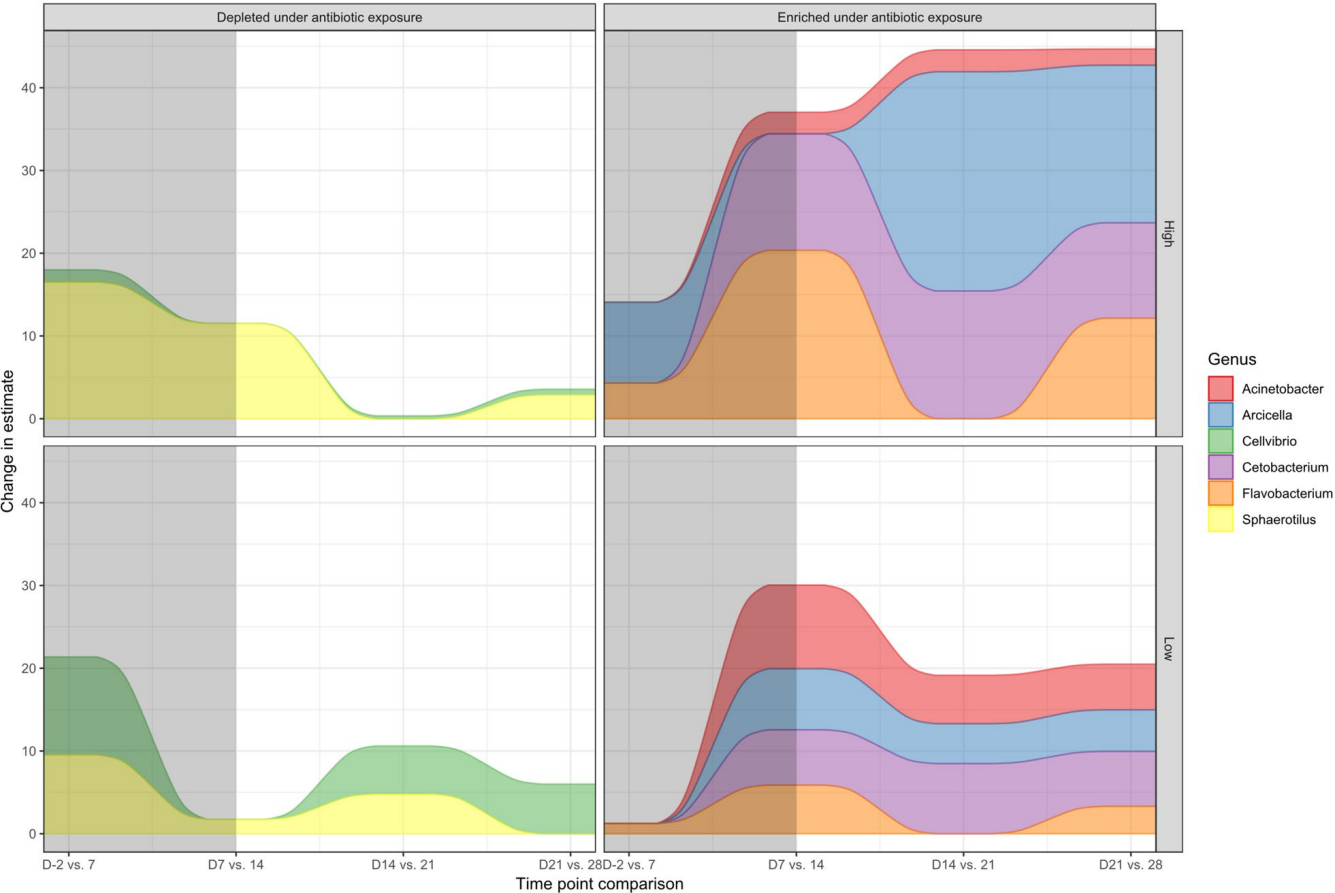
Differential abundance of the most differentially abundant microbial taxa over time in carp skin microbiomes under antibiotics exposure. The grey box indicates the period of antibiotic exposure. Timepoint comparison refers to when differential abundance is compared. Change in (model) estimates are predicted values of the change in taxa abundance derived from a linear mixed model (converted to relative abundance from raw counts). This shows the degree of change as relative abundance at the time points tested. Grey shading refers to comparison under antibiotic exposure. Full table available in Supplementary Table 4

The genera *Sphaerotilus* and *Arcicella* appeared to show strong associations with the beta diversity changes under antibiotic exposure (Fig. 4). The genus *Sphaerotilus* (phylum Proteobacteria) had a reduced relative abundance under high-concentration antibiotic exposure on Day 7 and Day 14 compared to pre-treatment levels (Day − 2 vs. 7, 14; model estimate − 9.01% and − 13.0% respectively, *p* < 0.05) (Supplementary Fig. 5A), and subsequently higher relative abundance during the period of depuration (Day 7 vs. 28; model estimate = 8.07%, Day 14 vs. 21, 28; model estimate 7.49% and 12.1% respectively, *p* < 0.05). A similar trend for *Sphaerotilus* was apparent for the low-concentration antibiotic exposure, although it took longer to return to pre-treatment levels, and was not significant (Supplementary Fig. 5B).

Contrasting with *Sphaerotilus*, the genus *Arcicella* (phylum Bacteroidota) displayed enrichment under high-concentration antibiotic exposure (Day − 2 vs. 7, 14; model estimate 13.1% and 16.4% respectively, *p* < 0.05) (Fig. 4), and during depuration *Arcicella* abundance was decreased (Day 14 vs. 21, 28; model estimate = − 13.4% and − 19.4% respectively, *p* < 0.05) (Supplementary Fig. 5A). This trend was also reflected in *Arcicella* abundance at a low concentration exposure with a lower model estimate, but this was not significant (Supplementary Fig. 5B).

### Differential abundance of microbiomes in tank water

Similar patterns in the microbiome for *Sphaerotilus* and *Arcicella* in response to antibiotic exposure in the carp skin, were seen in the water microbiome (Fig. 4). *Sphaerotilus* (Day − 2 vs. 14, 28; estimate − 18.9 and − 11.5 respectively, *p* < 0.05) was reduced with antibiotic exposure and increased during the depuration period, whereas *Arcicella* (Day 14 vs. 21; estimate − 22.2, *p* < 0.05) showed an increase in abundance compared to the pre-treatment levels and decreased during depuration (Supplementary Fig. 7).

### Short-term exposure to environmental concentrations of antibiotics does not alter ARG abundance or metabolic pathways in fish skin and water microbiomes

#### Antimicrobial resistant gene abundance following antibiotic exposure

**Fig. 5:**
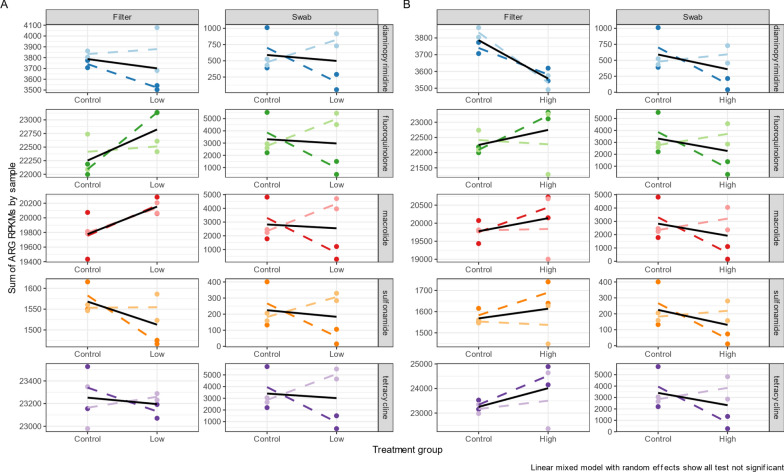
Total abundance of ARGs conferring resistances to either diaminopyrimidine (trimethoprim), fluoroquinolones (ciprofloxacin), macrolides (clarithromycin), sulphonamides (sulfamethoxazole), or tetracyclines (tetracycline). Abundance of ARG is normalised to Read per Kilobase Million (RPKM). Linear mixed model tests of significant changes in ARG abundance compared to either the low or high-concentration exposure groups. Different colours referred to different antibiotics, with lighter versions of each colour in different tanks within the same exposure group (replicates). **A** Low antibiotic concentration tanks. **B** High antibiotic concentration tanks

Following two weeks of antibiotic exposure (Day 14), we assessed the abundance of ARGs in the fish skin and tank water microbiomes in control and antibiotic treatment groups. A total of 1,622 unique ARGs were identified, conferring resistance to 40 different classes of antibiotics with the most prevalent resistance classes for tetracyclines, fluoroquinolones, and macrolides, in this order. Diaminopyrimidines and sulphonamides resistance genes were the 18th and 20th most abundant ARGs, respectively. No significant changes were detected in the abundance of ARGs conferring resistance to the five specifically assessed antibiotics or other antibiotic classes compared to the control group (Fig. 5). Similarly, analysis of individual ARG genes revealed no significant differences in abundance between treatment groups and the control.

### Metabolic profiles following antibiotic exposure

**Fig. 6:**
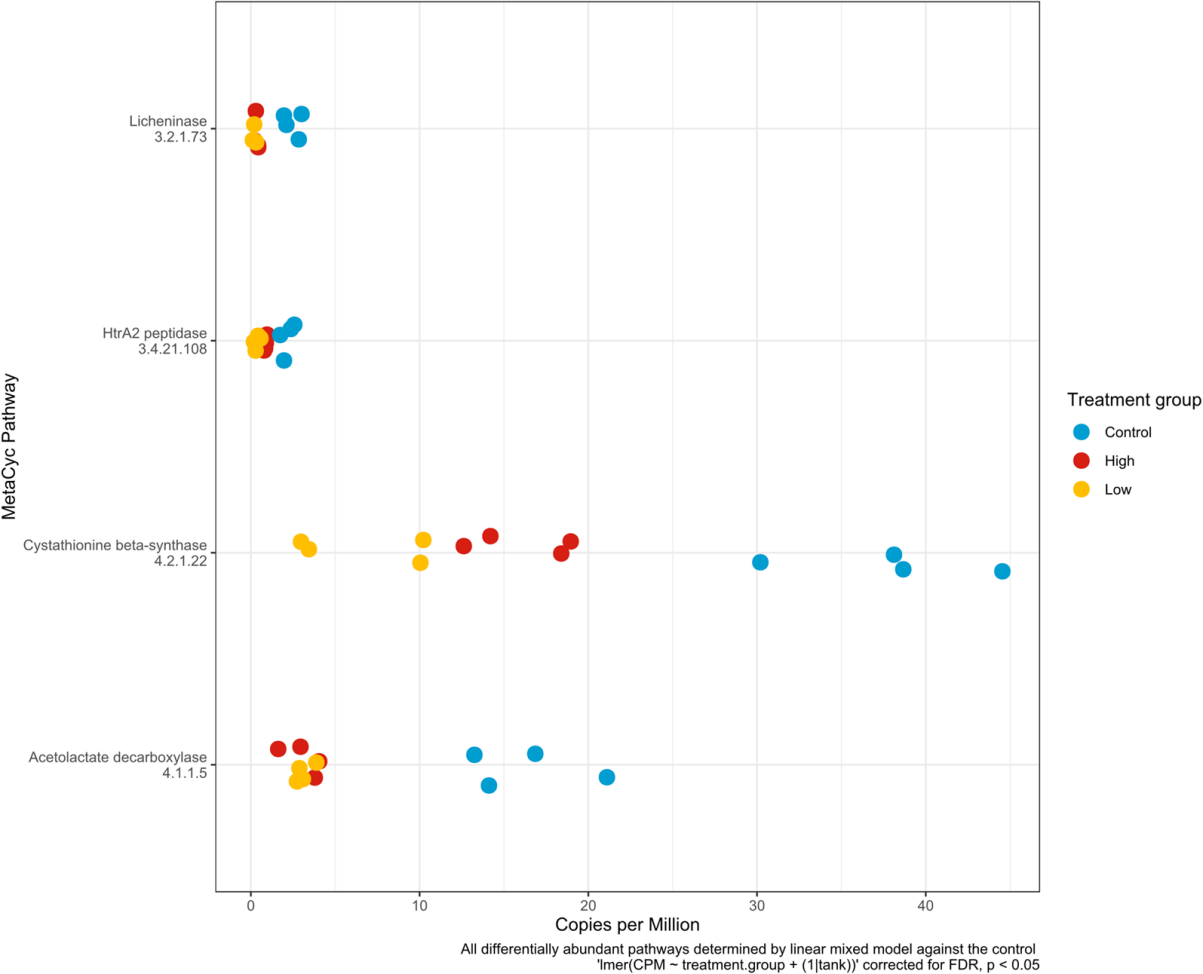
Differentially abundant MetaCyc pathways in tank water microbiomes after 14 days of antibiotic exposure. Differential abundance is determined using a linear mixed model corrected for false discovery rate using a *p* < 0.05 cutoff

Genes identified as part of a metabolic pathway within the carp skin and tank water microbiomes were determined after 14 days of antibiotic exposure. Carp skin microbiomes did not undergo any significant changes in the abundance of metabolic pathways, however, tank water microbiomes had significantly different metabolic profiles (Supplementary Fig. 8). Based on differential abundance, four metabolic pathways for licheninase (MetaCyc 3.2.1.73), HtrA2 peptidase (MetaCyc 3.4.21.108), cysthathionine beta-synthase (MetaCyc 4.2.1.22) and acetolactiate decarboxylase (MetaCyc 4.1.1.5) were all significantly depleted in low and high antibiotic concentration tank microbiomes, compared to the control (Fig. 6).

## Discussion

Using amplicon sequencing we show a relatively short (two-week) aqueous exposure to an environmentally relevant mixture of antibiotics resulted in a shift in beta diversity of carp skin and tank water microbiomes but with no effects on alpha diversity. Responses in skin microbiomes between individuals were consistent, with small observed differences in microbial community composition likely driven by changes in rare taxa abundance. Through metagenomic sequencing, we found no changes in the abundance of ARGs in either fish skin or tank water microbiomes. When comparing all detected genes in both fish skin and water microbiomes, there was evidence for reduced abundance of only four functional pathways in tank water microbiomes in both exposure groups compared to the controls.

### Responses in carp skin microbiomes to antibiotic exposure

A consideration when interpreting our findings is the reliance on relative abundance data. While this approach effectively characterises compositional shifts, it does not consider underlying changes in absolute microbial abundances. This limitation may obscure whether observed proportional changes are driven by factors such as differential resistance versus decline in overall microbial load. However, understanding the fundamental shifts in community proportions and structure, as revealed by relative abundance, provides valid insights into how microbiomes respond to stressors, such as antibiotics and this study focuses on interpreting these significant relative shifts in community composition.

The antibiotic exposure altered the overall composition of the carp skin bacterial communities, as measured by beta diversity, aligning with findings in other fish species including seabass [83, 84], tilapia [61] and yellowtail [53, 54] and even after the 2 weeks of depuration, exhibited only limited recovery, again mirroring observations in yellowtail, tilapia and seabass [53, 54, 61, 83, 84]. However, the antibiotic exposure had no apparent effect on the skin bacterial richness and evenness (alpha diversity), indicating that the total number and diversity of bacterial taxa on carp skin were not directly affected. Studies on the effects of orally dosed oxytetracycline (at 35 g/kg) on seabass (*Dicentrarchus labrax*) [84], and a combination of oxytetracycline (200 mg/kg) and both erythromycin and metronidazole (at 50 mg/kg) following disease on yellowtail (*Seriola lalandi*) [53, 54] similarly found no effect of the antibiotic exposure on alpha diversity. These findings contrast with studies on Nile tilapia [61] and mosquitofish (*Gambusia affinis)* [24] where skin microbiome alpha diversity was reduced for exposure to oxytetracycline and rifampicin respectively. However, these exposures were carried out at much higher, antibiotic exposure (therapeutic) levels (100 mg/kg for tetracycline and 25 µg/mL for rifampicin). Differences in the findings across the various studies are likely the result of differences in the fish species, and dosing method (differing concentrations, administration methods -orally versus through the water) as well as a result of the bioinformatic analysis methods applied (for example, centred log ratio (CLR) versus rarified count data)). A further explanation for differing reports on the effects of antibiotics on fish skin microbiome alpha diversity is that antibiotics may reduce bacterial populations without eliminating them, thus resulting in the same number of taxa present but with altered relative abundances. Illustrating this, in our study *Aeromonas* relative abundance was reduced by 36% (from 52.2 to 16.2%) following antibiotic exposure. It is also the case that in our study of carp skin microbiomes they may have contained highly abundant taxa resistant to antibiotics. For example, the genus *Arcicella* was seen to increase in relative abundance after antibiotic exposure (25.7% compared to 2.32% pre-treatment), potentially filling the niche left by other declining bacteria. Notably, the relative abundance of the genus *Sphaerotilus* increased from 8.0% at the end of antibiotic exposure to 25.6% after the depuration period, potentially as a consequence of additional available resources from those bacteria that had been suppressed by the antibiotic treatment and were less able (unable) to subsequently recover from the treatment.

The tank water microbiome community compositions were altered considerably more so than for the skin microbiome. Of all 2110 functional metabolic pathways identified in the carp skin and tank water microbiomes, only four appeared to be differentially abundant (all significantly depleted) for the water microbiome only (and none for the skin microbiome). This suggests that despite the changes seen in the water microbiome community compositions, the functional capacity likely remains largely unperturbed compared with the pre-exposure period. The greater shift in the community compositional change seen in the water microbiome may suggest that the water microbiomes are more sensitive to antibiotic exposure, with fish skin microbiomes potentially having a larger buffering capacity against chemical exposure.

In our study, the significant variation seen in the water microbiome beta diversity between tanks is similar to findings in studies on tilapia [61] and yellowtail [53, 54]. In these aforementioned studies, tank/pond, and even the sampling day are stronger influences on beta diversity than antibiotic exposure itself. These findings emphasise the dynamic nature of fish skin microbiomes, susceptibility to various and wide-ranging environmental influences and the complexity of dissecting the relative importance of antibiotic exposure, especially for environmentally relevant concentrations. This in turn clearly illustrates the value of including control tanks, that are essential for disentangling the effects of antibiotic exposure from those of other potential stressors affecting the system, particularly factors that may vary throughout the study.

### Skin bacterial phyla associated with antibiotic stress

Our study identified bacterial populations as potential indicators of antibiotic-induced dysbiosis in carp skin microbiomes, with enrichment occurring for Bacteroidetes and reduction for Proteobacteria. This has also been reported in sea bass skin microbiomes exposed to antibiotics [83]. This same pattern of response in these two phyla, however, was also seen for diseased seabass in the absence of an antibiotic challenge [84] which may suggest the possibility that these responses for Proteobacteria and Bacteroidetes reflect a more general stress response in fish skin microbiomes. No identical genera were found to be differential abundant within this study and in either seabass study by 83, 84), suggesting trends of genera decline or enrichment under antibiotic stress are more likely to be host species-specific.

Comparisons for the effects of antibiotic treatment on the skin microbiome at the bacterial genus level also appear to differ across different fish species, suggesting caution in interpreting specific bacteria as biomarkers for antibiotic effects across different fish species. Illustrating this, a study on tilapia fed oxytetracycline laced feed, identified an ASV from the *Aeromonas* genera and two from the Comamonadaceae family as significantly enriched [61], whereas in this study, *Sphaerotilus* (part of the Comamonadaceae family) *and Aeromonas* genus were depleted under antibiotic exposure. The study on tilapia also found that the genera *Cetobacterium* was significantly depleted under antibiotic exposure, whereas we found it was initially enriched under antibiotic exposure and subsequently depleted after the antibiotic exposure during the depuration phase of the study. Accounting for these differences is difficult but may reflect the influence of the differences between the studies relating to fish species (carp vs. tilapia), specific antibiotics used (oxytetracycline vs. our experiment’s antibiotic cocktail), antibiotic concentration, and antibiotic exposure route (feed vs. water). Given this, higher taxonomic classifications, such as phylum-level changes, might be more reliable as general bioindicators of antibiotic exposures on fish skin microbiomes.

It should also be noted that the enrichment of *Sphaerotilus* (within the Comamonadaceae family) that we saw in this study might not necessarily be related to antibiotic exposure only, as *Sphaerotilus* spp. are known to form prolific biofilms in organically enriched environments [58, 70]. A biofilm was seen to form in the exposure tanks during the experiment likely related to nutrient enrichment from the feeding of the fish (feed and faecal deposition), although an analysis to assess whether *Sphaerotilus* was indeed predominant in these biofilms was not carried out.

Enrichment of *Arcicella* emerged as a potential bioindicator for antibiotic exposure. *Arcicella* are free-living, gram-negative bacteria [69, 91] but their role(s) in fish skin microbiome function are unclear.

### Comparative responses between tank water and low-concentration exposure microbiomes suggest predictable antibiotic effects

Many of the trends observed in differential taxa abundance in the skin for the high-concentration concentration group were also seen in the low-concentration group, albeit to a lesser and they were statistically non-significant, and this may suggest that responses in carp skin microbiomes are potentially predictable. It is important to note, however, that some taxa, such as the env.OPS 17 family, were only differentially abundant in the low-concentration exposure group, albeit these were exceptions to the general findings. There were strong similarities also in the shifts in the microbiome assemblages between the skin and tank water for the low concentration antibiotic exposure group which may suggest similar mechanisms driving these changes.

The strong similarity between the tank water and fish skin microbiomes for all treatment groups is likely, at least in part due to the tank water used for their exposures, which was dechlorinated tap water. This would contain a very limited bacterial content and the bacteria that shed from the carp skin into the relatively small tank volumes, would in turn likely quickly dominate the water microbiome. This would likely be different for fish held in natural pondwaters. The high degree of similarity between the fish skin and tank water communities also suggests that biofilms and other secondary tank reservoirs may serve as a source of bacteria that repopulate the fish skin during the depuration period. This finding aligns with a study of aquacultural systems, where tank infrastructures closely resembled the tank water microbiomes (and less so the fish mucosal surface microbiome) [64]. This was also true for tank biofilms colonised by probiotics [16], which may serve as a source of bacteria for recolonising the fish skin during the depuration period. This highlights the potential importance of the external environment and secondary microbial reservoirs for fish mucosal surface microbiome recovery after exposure to antibiotics and other microbiome disrupting stressors. Further investigations might seek to better understand the specific role(s) of biofilms and other reservoirs external to the fish in facilitating fish microbiome recolonisation following stressing events. This discussion emphasises the need for a degree of caution when seeking to extrapolate for the effects of antibiotics under controlled laboratory conditions and those that may occur in the wild in natural environments and including in aquaculture systems where there are likely to be more diverse (and unique) water microbiomes that may differ in both their responses to antibiotic exposures and their interrelationships with the fish skin microbiome for effects on and recovery from antibiotics exposures.

### Absence of antibiotic effects on antimicrobial resistance gene abundance in carp skin or tank water

Contrary to our initial hypothesis, a notable finding of this study was the absence of statistically significant increases in the abundance of ARGs conferring resistance to any of the antibiotic classes employed (diaminopyrimidine, fluoroquinolones, macrolides, sulphonamides, tetracyclines) or any other antibiotic class within the fish skin microbiome following two weeks of exposure to environmentally relevant antibiotic cocktails. This was somewhat unexpected, given the inherent selective pressure antibiotics exert, and aligns with the broader observation of relatively limited impacts on overall community richness and functional metabolic pathways in this specific experimental context. Several factors could contribute to this apparent resilience or lack of detectable ARG response. The relatively short two-week exposure duration might be insufficient to drive substantial ARG selection or horizontal gene transfer on a dynamic surface like fish skin, potentially requiring longer periods for such effects to manifest clearly.

In other studies, on fish, antibiotic treatments have been shown to increase corresponding ARGs. In zebrafish (*Danio rerio*), for example, exposure to 1 or 5 µg/L of sulfamethoxazole or oxytetracycline (the high concentration exposure in this study for sulfamethoxazole was ~ 1.01 µg/L and for oxytetracycline ~ 2.09 µg/L) for 120 days resulted in a significant increase in corresponding ARGs within the gut microbiomes [42]. In Nile tilapia too exposure to oxytetracycline (at 100 mg/kg/body weight/day) for 8 days increased the tetA resistance gene [76]. Both of these studies however were on the gut microbiome and the development of ARGs is likely affected not only by the antibiotic exposure level but also by the tissue environment/body compartment due to nutritional (and other) factors [12, 81, 94], although this has not been studied in fish.

It should also be recognised that the source of the fish may have an effect on the development of ARGs. Fish derived from farms with no previous antibiotic exposure (or with little antibiotic pressure), as in the case of the carp in this study, may have fewer ARGs and a longer time under the antibiotic pressure is required to induce mutations conferring resistance. ARG abundance also may not directly reflect gene expression and some ARGs may be transcribed at higher levels under antibiotic pressure even if their overall abundance in the microbiome remains relatively unchanged. Future studies might address these questions as a possibility through transcriptomic analysis of fish skin microbiomes under antibiotic pressure.

While these findings under environmentally relevant concentrations suggest the threshold or timescale for ARG proliferation may vary depending on the host, tissue, and environmental context, they need to be interpreted cautiously. This study highlights the context-dependency of antibiotic impacts and does not negate the broader, well-established concerns regarding ARG selection by antibiotic pollution across diverse ecosystems. Future investigations employing longer exposure durations, analysing different environmental compartments (like sediment or gut), and potentially using higher-sensitivity detection methods are needed to fully delineate environmental ARG risks.

### Concluding statement

We show a relatively short-term exposure to a mixture of antibiotics at environmentally relevant concentrations can alter the balance of the skin microbial communities in the skin of fish. The findings of enrichment of the genus *Arcicella* (Proteobacteria) and depletion of *Sphaerotilus* (Bacteroidetes) may provide potential bioindicators of antibiotic exposure. The study also indicates that the tank environment and microbial genera may affect the recolonisation and recovery dynamics of the skin microbiomes post antibiotic exposure. Further research is necessary to elucidate the long-term consequences of these microbial community shifts on fish health, resilience, and potential for antibiotic resistance development. The findings from this study warrant further investigations into the effects of chronic, low-concentration exposure to antibiotics on the functional capacity of the altered microbiomes and how this affects fish health. This is especially important in aquaculture practices where there is the need to ensure that antibiotics do not compromise the longer-term health of the fish, as well as to minimise the environmental impact of antibiotics.

## Supplementary Information

Additional file1

Additional file1

## Data Availability

The data for this study have been deposited in the European Nucleotide Archive (ENA) at EMBL-EBI under accession number PRJEB82662. All bioinformatic processes and subsequent analyses can be found online at: https://github.com/ash-bell/fish_skin_antibiotic_exposure.
