# Supplementary material for: Impacts of environmentally relevant concentrations of antibiotic cocktails on the skin microbiome of Eurasian carp (Cyprinus carpio)
Source: Anim Microbiome. 2025 Jul 8;7:73. doi: 10.1186/s42523-025-00434-8 (PMC12239446; doi:10.1186/s42523-025-00434-8)
Supplement: Supplementary file 1 — Additional file1 [file 42523_2025_434_MOESM1_ESM.docx]

# Supplementary Figures


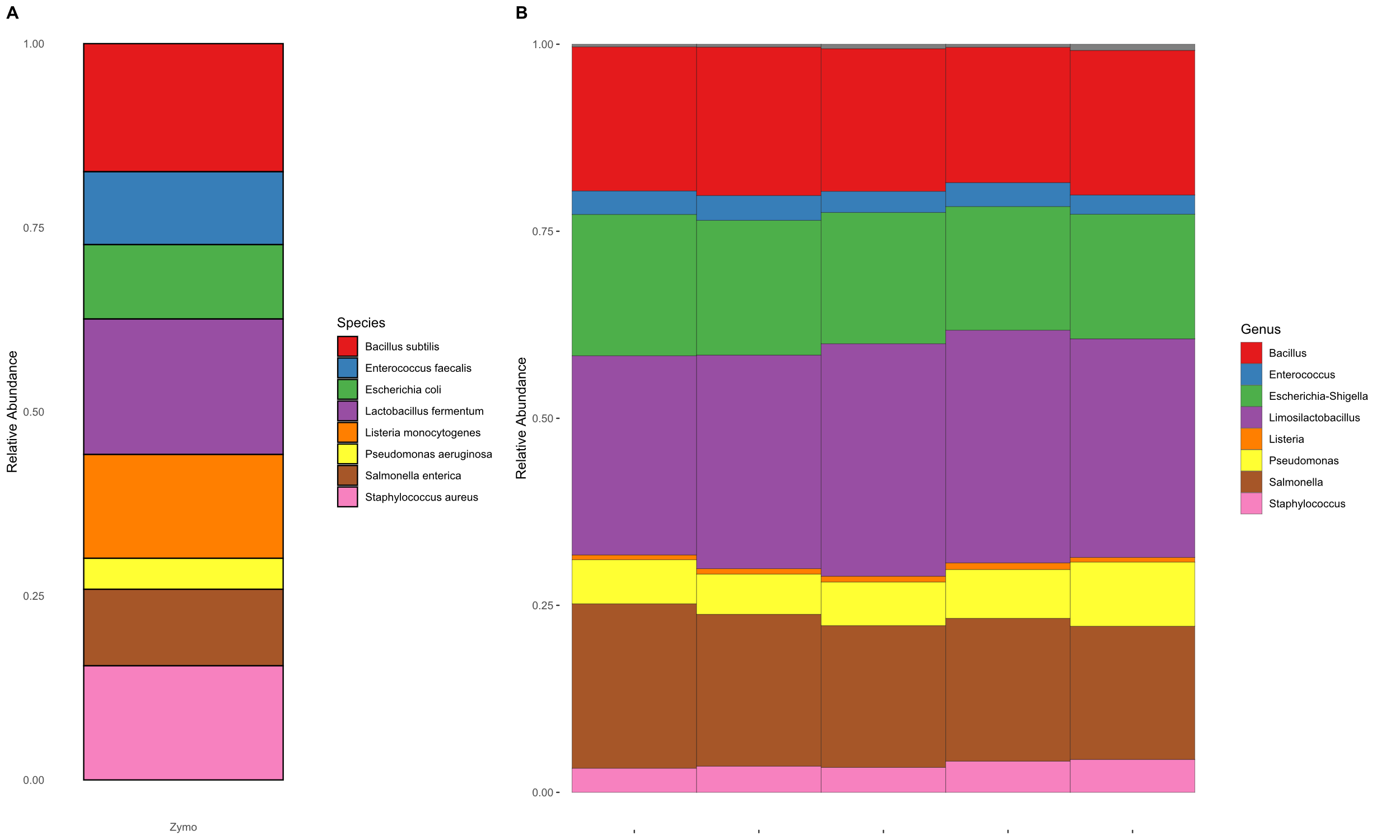
Supplementary Figure 1 Positive control samples consisting of B) 5 ZymoBIOMICS Microbial Community Standard versus the A) expected proportion of taxa showing the effectiveness and evenness of DNA extraction methods used within this study.


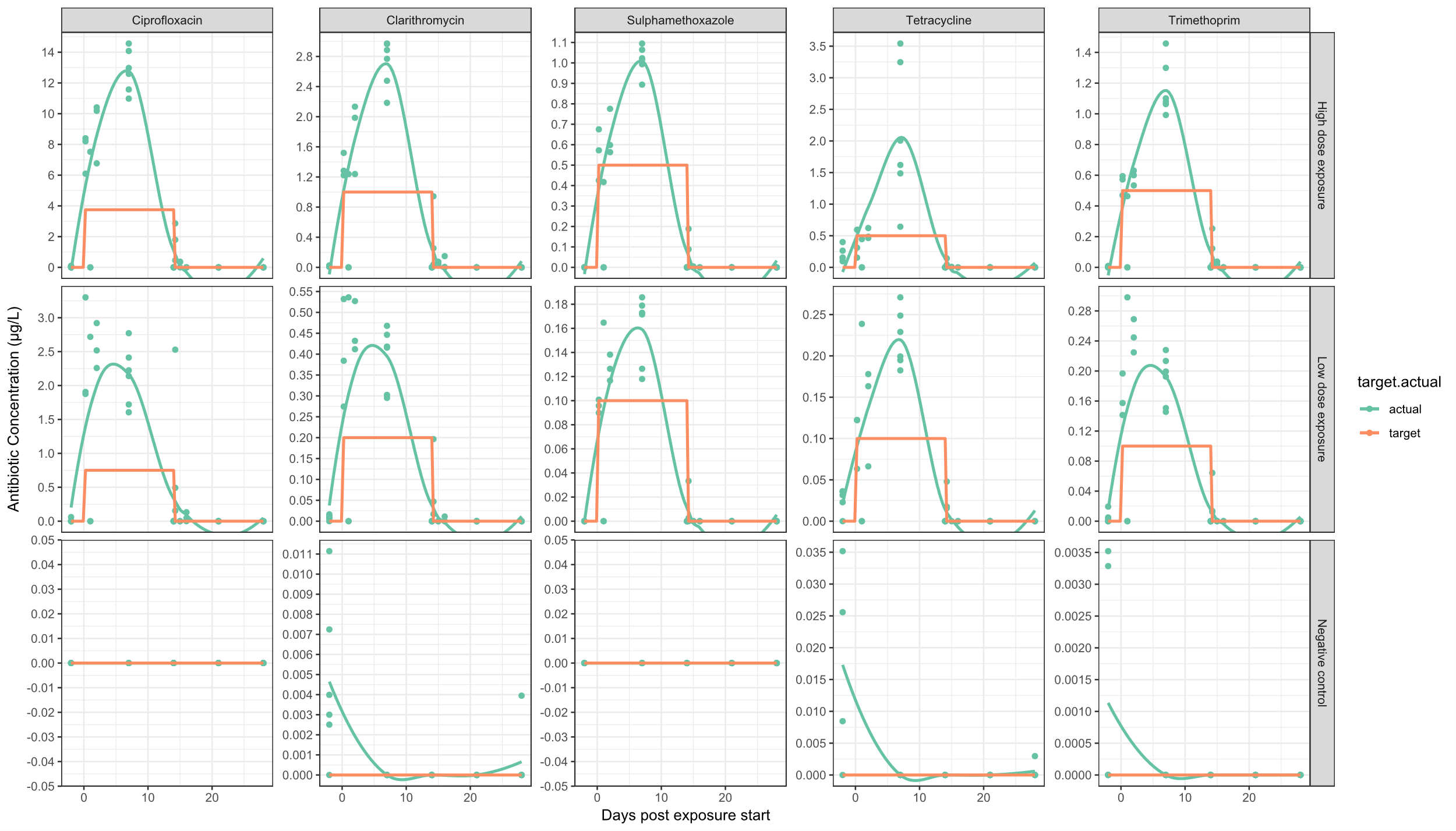


Supplementary Figure 2 The expected and actual antibiotic exposure concentration observed within this experiment split by the five antibiotics used and the treatment they are part of.


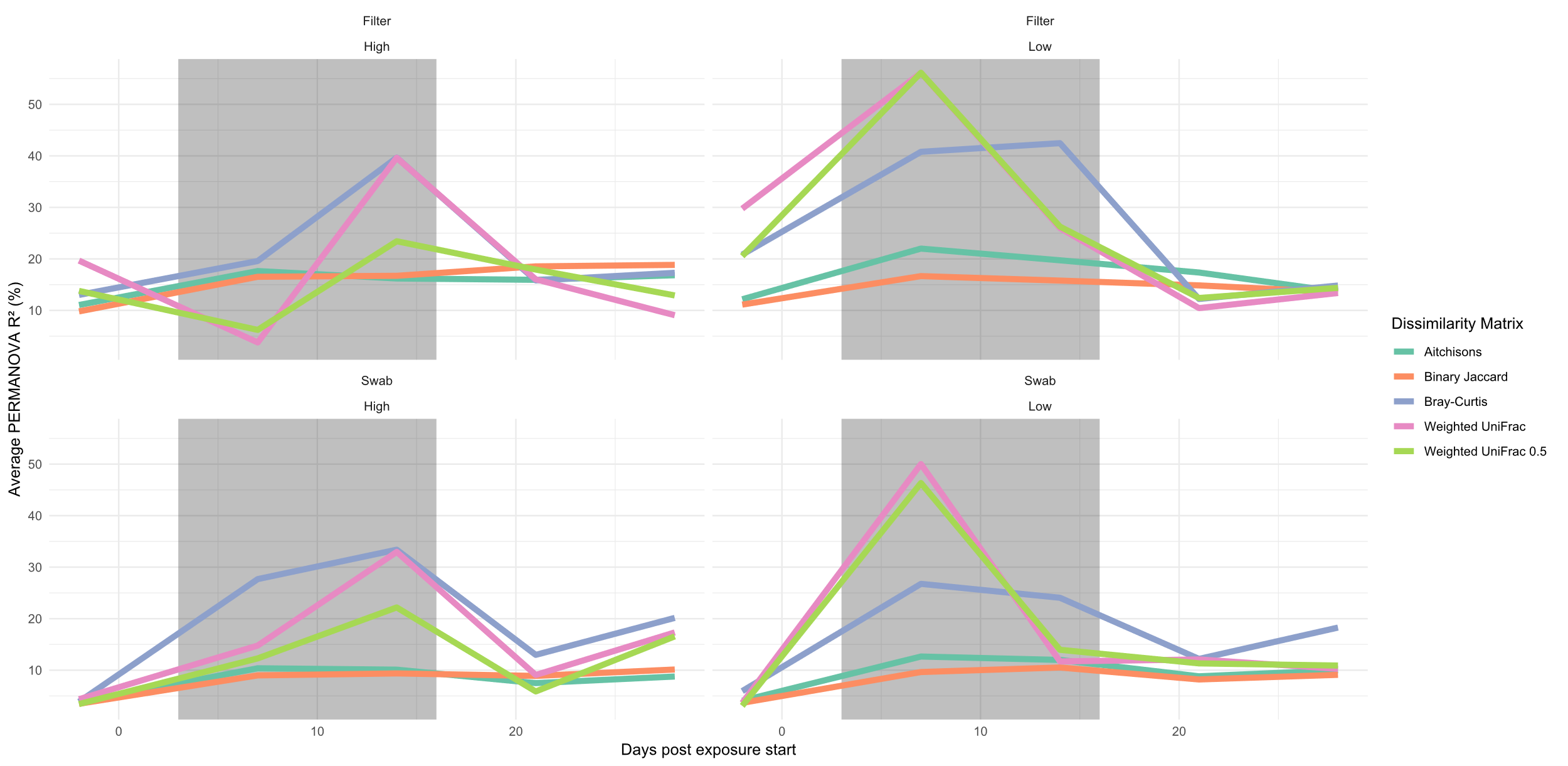


Supplementary Figure 3 Different beta diversities used to measure the difference in beta diversity of carp skin and tank water microbiomes over time. The grey box indicates the period of antibiotic exposure.


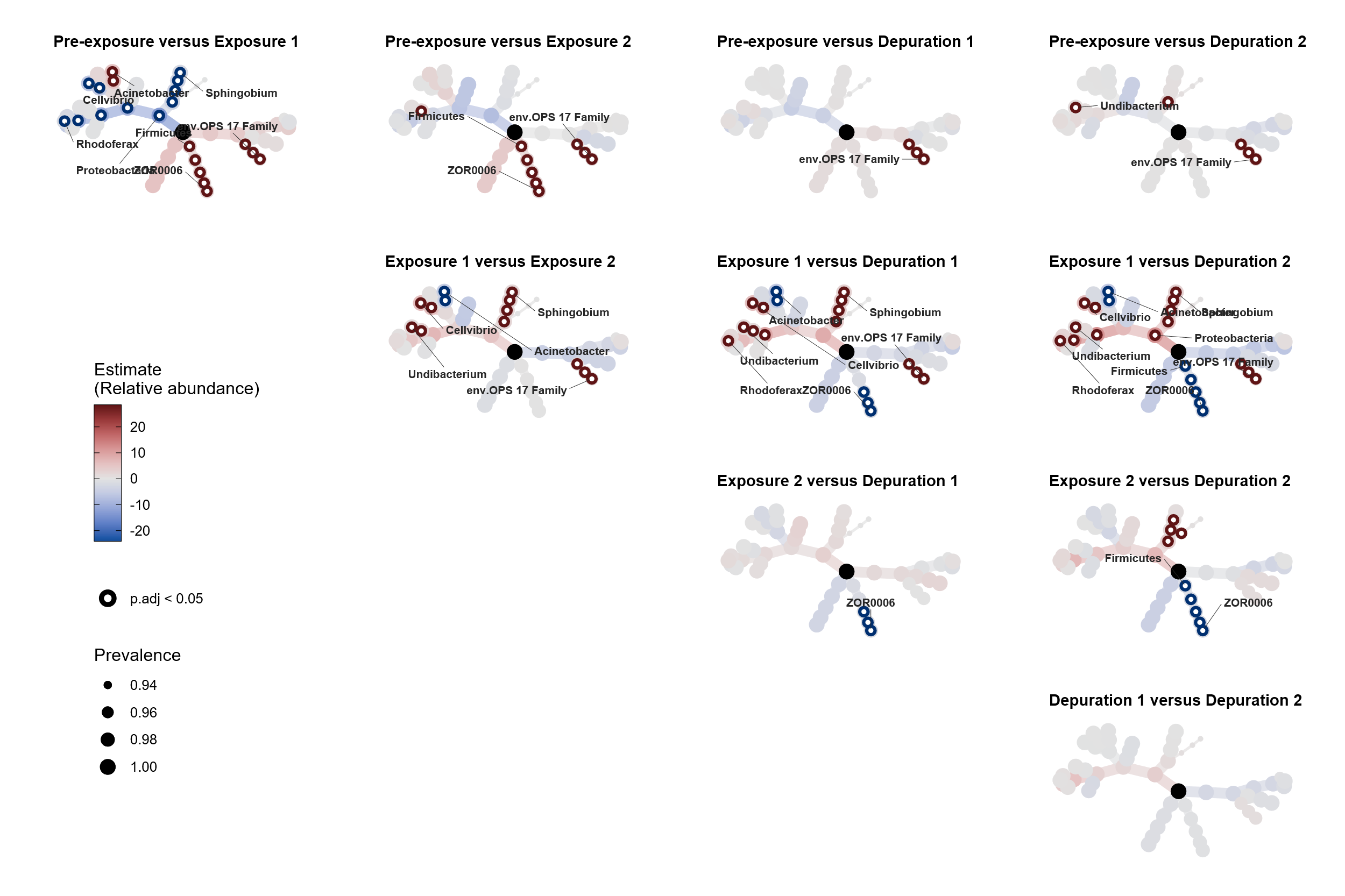


**A**


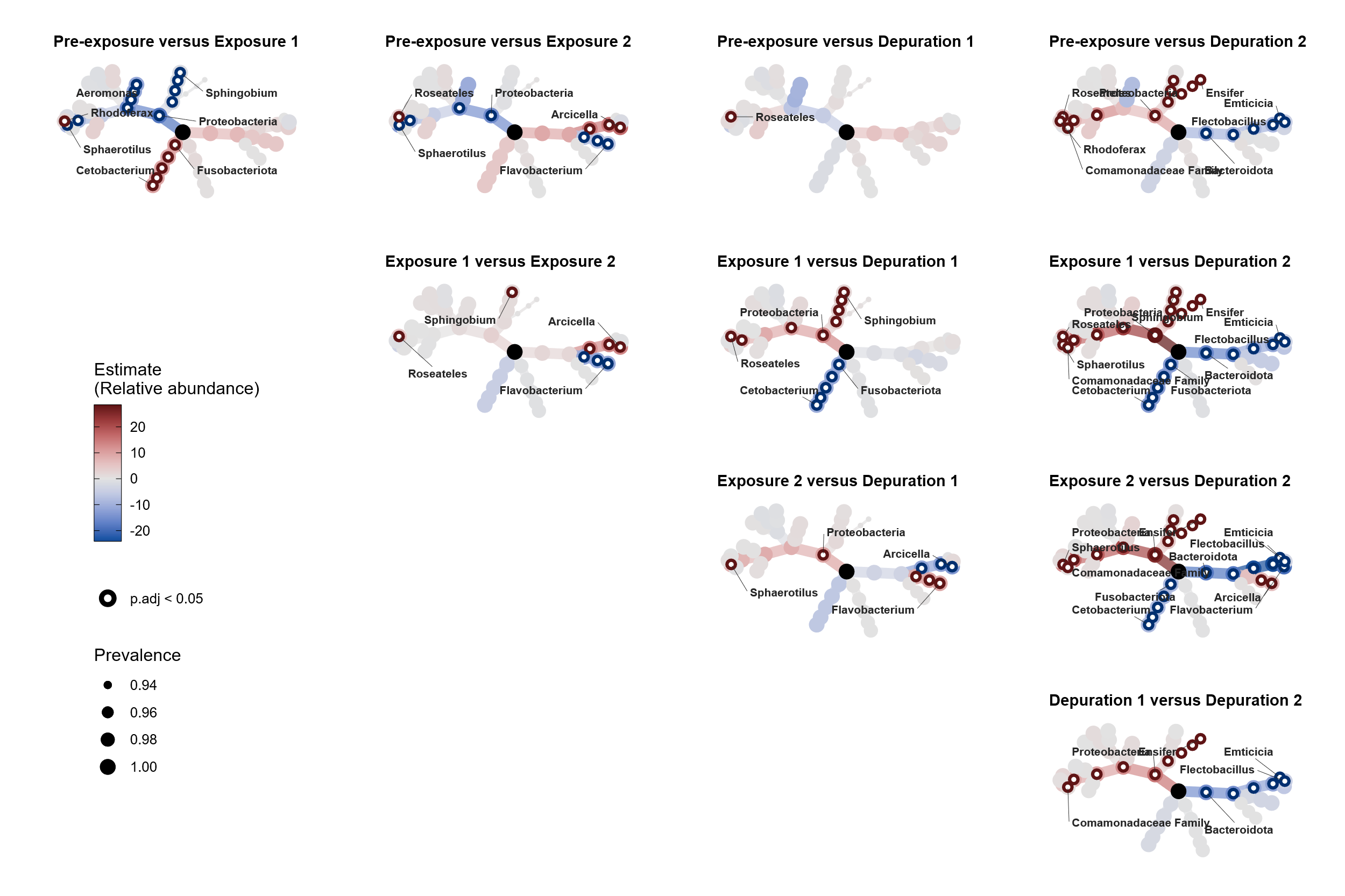


**B**

Supplementary Figure 4 Taxonomic trees showing bacterial taxa significantly altered in abundance within the fish skin microbiome during a two week antibiotic exposure and subsequent two week depuration. Panels depict responses to (A) low concentration and (B) high concentration antibiotic exposure over two weeks, followed by a two-week depuration period. Taxa significantly enriched (relative to the non-exposed control group) are highlighted in red, while significantly depleted taxa are highlighted in blue. Differential abundance was determined using raw counts, but data are visualised here as relative abundance. Time points represent: Pre-exposure (2 days prior), Exposure 1 (7 days exposure), Exposure 2 (14 days exposure), Depuration 1 (7 days recovery post-exposure), and Depuration 2 (14 days recovery post-exposure). Only taxa identified at the genus or phylum level, present in >1% of samples, and showing significant abundance changes after False Discovery Rate (FDR) correction are displayed. Comprehensive results can be found in (Supplementary Table 7).


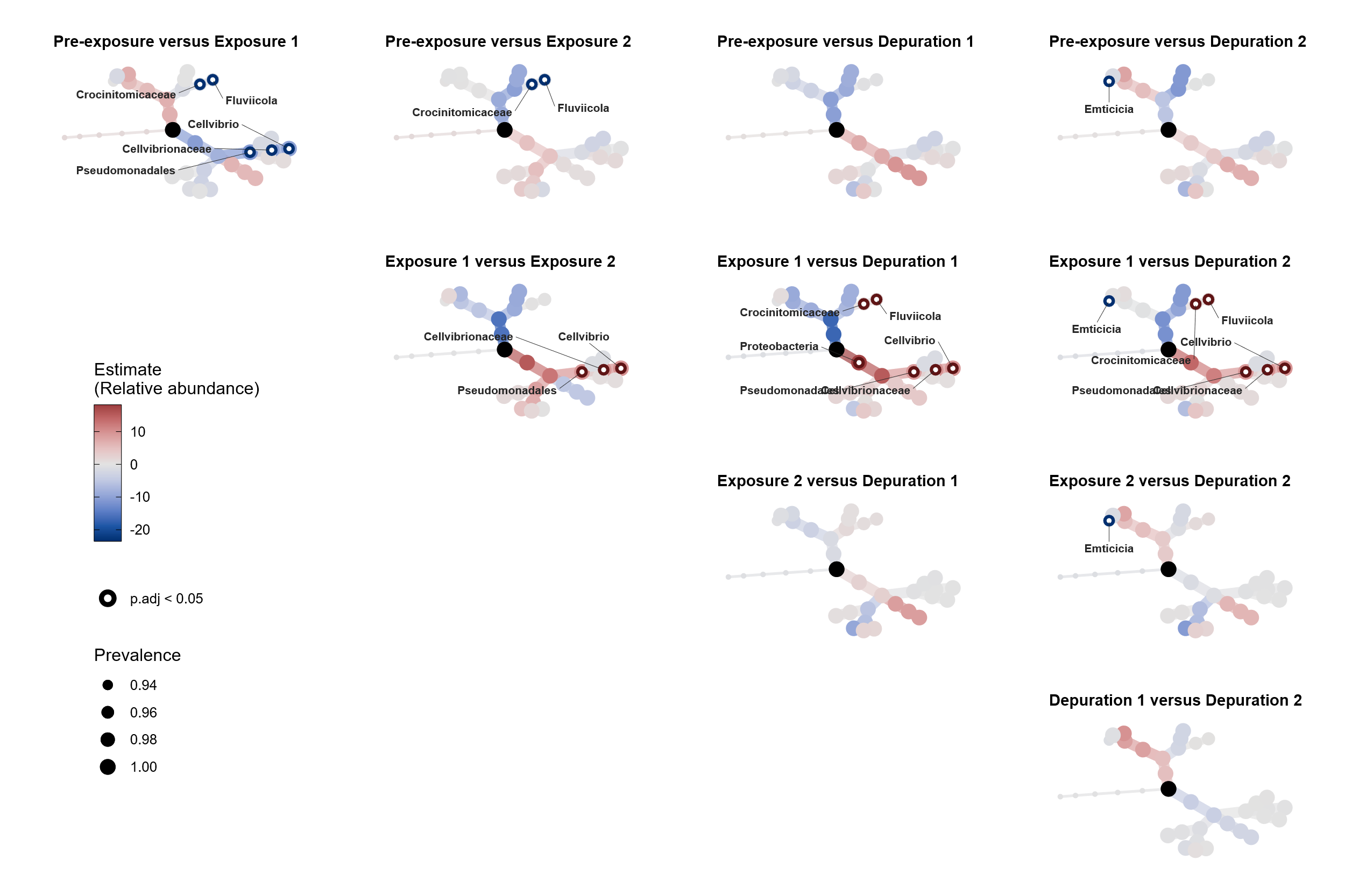


**A**


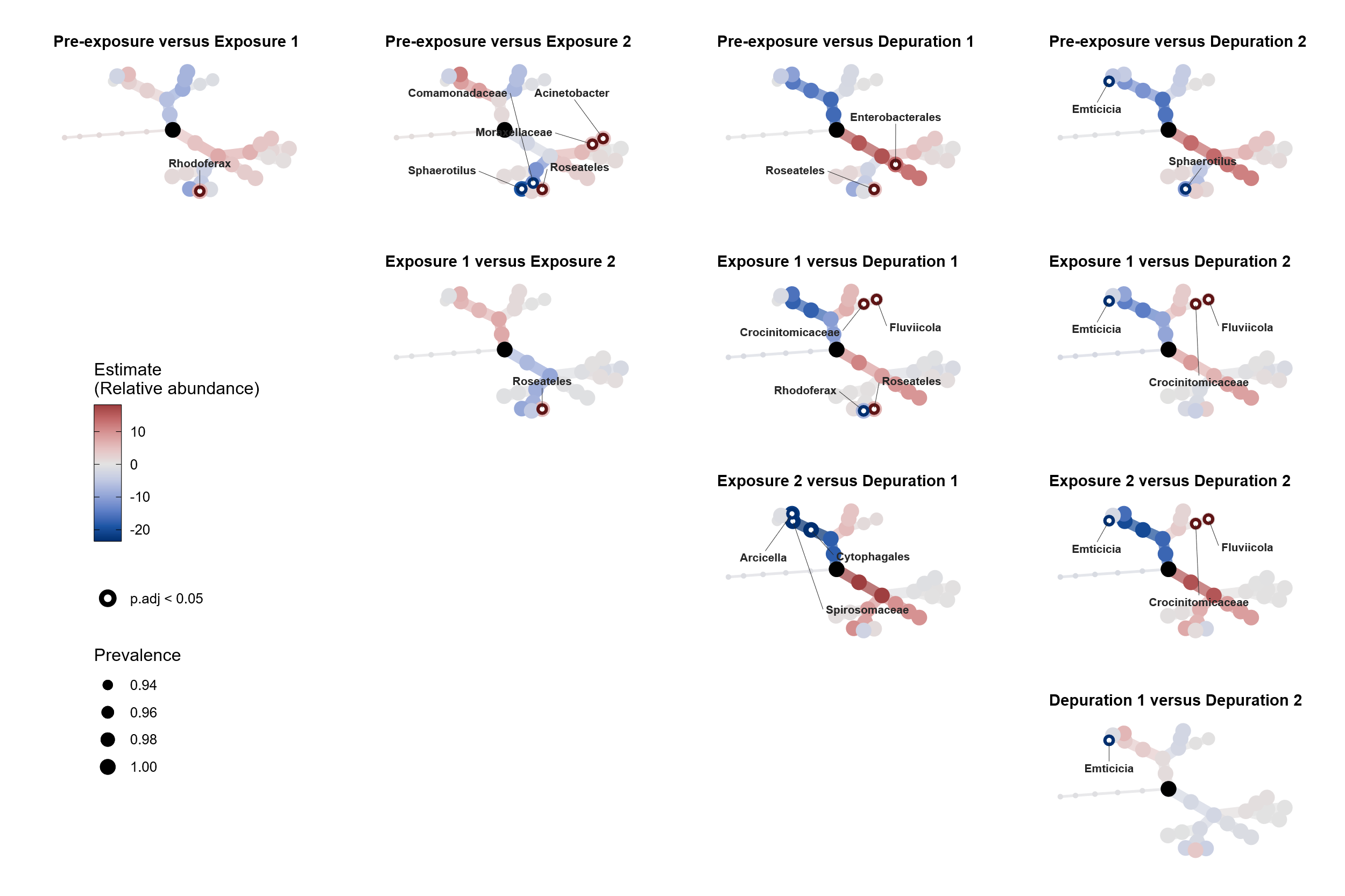


**B**

Supplementary Figure 5 Taxonomic trees showing bacterial taxa significantly altered in abundance within tank water microbiomes dring a two week antibiotic exposure and subsequent two week depuration. Panels depict responses to (A) low concentration and (B) high concentration antibiotic exposure over two weeks, followed by a two-week depuration period. Taxa significantly enriched (relative to the non-exposed control group) are highlighted in red, while significantly depleted taxa are highlighted in blue. Differential abundance was determined using raw counts, but data are visualised here as relative abundance. Time points represent: Pre-exposure (2 days prior), Exposure 1 (7 days exposure), Exposure 2 (14 days exposure), Depuration 1 (7 days recovery post-exposure), and Depuration 2 (14 days recovery post-exposure). Only taxa identified at the genus or phylum level, present in >1% of samples, and showing significant abundance changes after False Discovery Rate (FDR) correction are displayed. Comprehensive results can be found in (Supplementary Table 7).


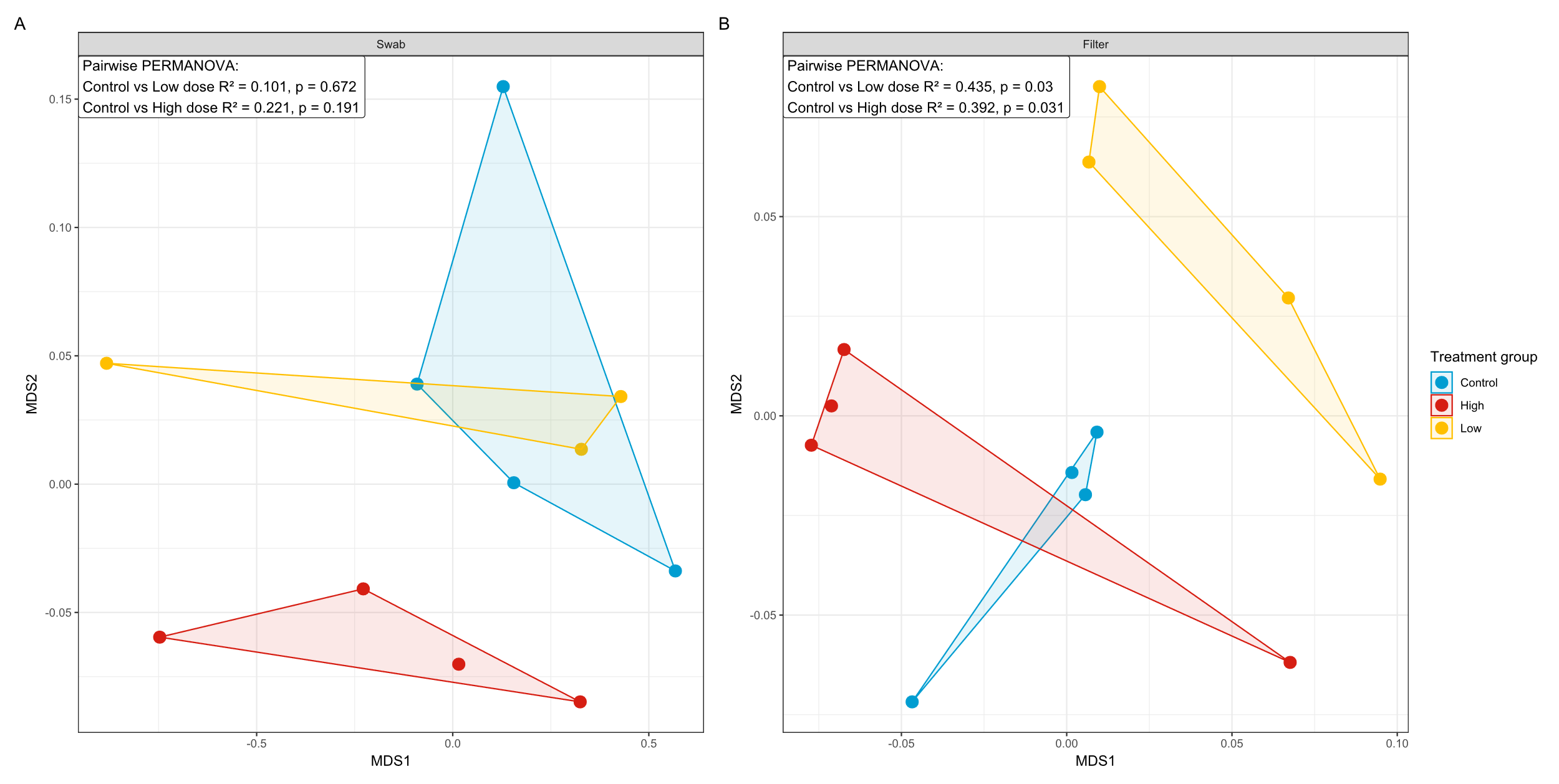


Supplementary Figure 6 Bacterial metabolic profiles of carp skin and tank water microbiomes under 14 days of antibiotic exposure. Metabolic profiles are transformed using a Bray-Curtis dissimularity matrix and visualed using an MDS ordination. Significantly different metabolic profiles from each treatment group are determined usinga a pairwise PERMANOVA. Samples are split by fish skin microbiome (swab) and tank water microbiome (filter).

# Supplementary Tables

Supplementary Table 1 Surface water antibiotic concentrations from various studies. ^1^WWTP effluent ^2^Surface water

| Antibiotic (Class) | (Wang *et al.*, 2020)**^1^** | (Booth, Aga and Wester, 2020)**^2^** | **UKWIR Chemicals Investigation Database^2^** | (Danner *et al.*, 2019)**^2^** | (Bengtsson-Palme and Larsson, 2016)**^1^** | (Sanseverino *et al.*, 2018)**^2^** | Median |  | Low dose cocktail | High dose cocktail |
| --- | --- | --- | --- | --- | --- | --- | --- | --- | --- | --- |
| Concentration | c | | | | | | | | | |
| Ciprofloxacin (Fluoroquinolones) | 0.096 | 383.4 | 0.123 | 2.149571 | 0.742 | 0.8 | 0.74 |  | 0.75 | 3.75 |
| Clarithromycin (Macrolide) | 0.1939 | 0.1 | 0.145674 | 0.5432 | 0.61 | 0.7 | 0.19 |  | 0.20 | 1.0 |
| Sulfamethoxazole (Sulphonamides) | 0.097 | 0.1 | 0.025209 | 9.594429 | 0.964 | 0.8 | 0.10 |  | 0.10 | 0.5 |
| Trimethoprim | 0.138 | 0 | 0.008471 | 2.664789 | 2.4 | 0.1 | 0.14 |  | 0.10 | 0.5 |
| Tetracycline | 0.0345 | 0 | - | 0.172889 | 0.62 | 0.4 | 0.10 |  | 0.10 | 0.5 |
| MEDIAN | 0.097 | 0.1 | 0.074104 | 2.149571 | 0.742 | 0.7 | 0.10 |  | 0.15 | 0.75 |

Supplementary Table 2 16S amplicon forward and reverse primer sequences and combinations used within this study.

| Well Position | Forward Read Name | Forward Read Sequence | Reverse Read Name | Reverse Read Sequences |
| --- | --- | --- | --- | --- |
| A1 | 515fcbc61 | GTGGAGTCTCATGTGTGYCAGCMGCCGCGGTAA | 806rcbc147 | ACAACACTCCGACCGGACTACNVGGGTWTCTAAT |
| A2 | 515fcbc160 | AGTGTTTCGGACGTGTGYCAGCMGCCGCGGTAA | 806rcbc220 | GTCGTCCAAATGCCGGACTACNVGGGTWTCTAAT |
| A3 | 515fcbc151 | AGCTATGTATGGGTGTGYCAGCMGCCGCGGTAA | 806rcbc202 | GTCTTCAGCAAGCCGGACTACNVGGGTWTCTAAT |
| A4 | 515fcbc38 | ACCATAGCTCCGGTGTGYCAGCMGCCGCGGTAA | 806rcbc247 | AGGTCCAAATCACCGGACTACNVGGGTWTCTAAT |
| A5 | 515fcbc69 | TATCGACACAAGGTGTGYCAGCMGCCGCGGTAA | 806rcbc132 | TCCTCGAGCGATCCGGACTACNVGGGTWTCTAAT |
| A6 | 515fcbc152 | ACGGGTCATCATGTGTGYCAGCMGCCGCGGTAA | 806rcbc124 | CCTGCGAAGTATCCGGACTACNVGGGTWTCTAAT |
| A7 | 515fcbc52 | ACTCACAGGAATGTGTGYCAGCMGCCGCGGTAA | 806rcbc244 | GAGACGTGTTCTCCGGACTACNVGGGTWTCTAAT |
| A8 | 515fcbc34 | TTGCGTTAGCAGGTGTGYCAGCMGCCGCGGTAA | 806rcbc115 | TACCGAAGGTATCCGGACTACNVGGGTWTCTAAT |
| A9 | 515fcbc142 | GCATAGCATCAAGTGTGYCAGCMGCCGCGGTAA | 806rcbc4 | TGGTCAACGATACCGGACTACNVGGGTWTCTAAT |
| A10 | 515fcbc146 | AGAGTCTTGCCAGTGTGYCAGCMGCCGCGGTAA | 806rcbc167 | CGCTACAACTCGCCGGACTACNVGGGTWTCTAAT |
| A11 | 515fcbc23 | GCAACACCATCCGTGTGYCAGCMGCCGCGGTAA | 806rcbc135 | AGCAACATTGCACCGGACTACNVGGGTWTCTAAT |
| A12 | 515fcbc138 | GTAGAGGTAGAGGTGTGYCAGCMGCCGCGGTAA | 806rcbc153 | GAAACATCCCACCCGGACTACNVGGGTWTCTAAT |
| B1 | 515fcbc145 | TGAACCCTATGGGTGTGYCAGCMGCCGCGGTAA | 806rcbc111 | AGCTGTCAAGCTCCGGACTACNVGGGTWTCTAAT |
| B2 | 515fcbc15 | GAATACCAAGTCGTGTGYCAGCMGCCGCGGTAA | 806rcbc146 | AGAGTCTTGCCACCGGACTACNVGGGTWTCTAAT |
| B3 | 515fcbc66 | CTCACCTAGGAAGTGTGYCAGCMGCCGCGGTAA | 806rcbc83 | GTTCTCTTCTCGCCGGACTACNVGGGTWTCTAAT |
| B4 | 515fcbc125 | TTCTCTCGACATGTGTGYCAGCMGCCGCGGTAA | 806rcbc246 | TATGCCAGAGATCCGGACTACNVGGGTWTCTAAT |
| B5 | 515fcbc154 | CGTACTCTCGAGGTGTGYCAGCMGCCGCGGTAA | 806rcbc66 | CTCACCTAGGAACCGGACTACNVGGGTWTCTAAT |
| B6 | 515fcbc150 | ACGCGAACTAATGTGTGYCAGCMGCCGCGGTAA | 806rcbc49 | GGCCAGTTCCTACCGGACTACNVGGGTWTCTAAT |
| B7 | 515fcbc100 | GATGTATGTGGTGTGTGYCAGCMGCCGCGGTAA | 806rcbc125 | TTCTCTCGACATCCGGACTACNVGGGTWTCTAAT |
| B8 | 515fcbc30 | AGATTGACCAACGTGTGYCAGCMGCCGCGGTAA | 806rcbc270 | GATCTCTGGGTACCGGACTACNVGGGTWTCTAAT |
| B9 | 515fcbc62 | GCTCGAAGATTCGTGTGYCAGCMGCCGCGGTAA | 806rcbc35 | TACGAGCCCTAACCGGACTACNVGGGTWTCTAAT |
| B10 | 515fcbc113 | TACTCGGGAACTGTGTGYCAGCMGCCGCGGTAA | 806rcbc186 | CTGATCCATCTTCCGGACTACNVGGGTWTCTAAT |
| B11 | 515fcbc123 | ATGTGTGTAGACGTGTGYCAGCMGCCGCGGTAA | 806rcbc221 | CAACGTGCTCCACCGGACTACNVGGGTWTCTAAT |
| B12 | 515fcbc97 | TGTGCGATAACAGTGTGYCAGCMGCCGCGGTAA | 806rcbc173 | AACGTAGGCTCTCCGGACTACNVGGGTWTCTAAT |
| C1 | 515fcbc99 | GCCTAGCCCAATGTGTGYCAGCMGCCGCGGTAA | 806rcbc86 | GTTGTTCTGGGACCGGACTACNVGGGTWTCTAAT |
| C2 | 515fcbc46 | TGTGAATTCGGAGTGTGYCAGCMGCCGCGGTAA | 806rcbc8 | ATCCTTTGGTTCCCGGACTACNVGGGTWTCTAAT |
| C3 | 515fcbc19 | CCAATACGCCTGGTGTGYCAGCMGCCGCGGTAA | 806rcbc31 | AGTTACGAGCTACCGGACTACNVGGGTWTCTAAT |
| C4 | 515fcbc158 | GATCACGAGAGGGTGTGYCAGCMGCCGCGGTAA | 806rcbc175 | TCGTCAAACCCGCCGGACTACNVGGGTWTCTAAT |
| C5 | 515fcbc103 | GCGAGCGAAGTAGTGTGYCAGCMGCCGCGGTAA | 806rcbc108 | GCACACCTGATACCGGACTACNVGGGTWTCTAAT |
| C6 | 515fcbc6 | GTCGTGTAGCCTGTGTGYCAGCMGCCGCGGTAA | 806rcbc6 | GTCGTGTAGCCTCCGGACTACNVGGGTWTCTAAT |
| C7 | 515fcbc112 | GAGAGCAACAGAGTGTGYCAGCMGCCGCGGTAA | 806rcbc87 | GGACTTCCAGCTCCGGACTACNVGGGTWTCTAAT |
| C8 | 515fcbc63 | AGGCTTACGTGTGTGTGYCAGCMGCCGCGGTAA | 806rcbc89 | CTGCTATTCCTCCCGGACTACNVGGGTWTCTAAT |
| C9 | 515fcbc17 | TAACGTGTGTGCGTGTGYCAGCMGCCGCGGTAA | 806rcbc26 | AGTCGTGCACATCCGGACTACNVGGGTWTCTAAT |
| C10 | 515fcbc106 | TCTTGGAGGTCAGTGTGYCAGCMGCCGCGGTAA | 806rcbc219 | TGGTTGGTTACGCCGGACTACNVGGGTWTCTAAT |
| C11 | 515fcbc94 | TTGGCTCTATTCGTGTGYCAGCMGCCGCGGTAA | 806rcbc234 | CTCGTGAATGACCCGGACTACNVGGGTWTCTAAT |
| C12 | 515fcbc108 | GCACACCTGATAGTGTGYCAGCMGCCGCGGTAA | 806rcbc172 | CACTGGTGCATACCGGACTACNVGGGTWTCTAAT |
| D1 | 515fcbc117 | GTATTTCGGACGGTGTGYCAGCMGCCGCGGTAA | 806rcbc105 | ACTTGGTGTAAGCCGGACTACNVGGGTWTCTAAT |
| D2 | 515fcbc12 | TGCATACACTGGGTGTGYCAGCMGCCGCGGTAA | 806rcbc80 | TAGTATGCGCAACCGGACTACNVGGGTWTCTAAT |
| D3 | 515fcbc140 | GGTTATTTGGCGGTGTGYCAGCMGCCGCGGTAA | 806rcbc17 | TAACGTGTGTGCCCGGACTACNVGGGTWTCTAAT |
| D4 | 515fcbc80 | TAGTATGCGCAAGTGTGYCAGCMGCCGCGGTAA | 806rcbc144 | TTAGAGCCATGCCCGGACTACNVGGGTWTCTAAT |
| D5 | 515fcbc28 | CGAGGGAAAGTCGTGTGYCAGCMGCCGCGGTAA | 806rcbc16 | GTAGATCGTGTACCGGACTACNVGGGTWTCTAAT |
| D6 | 515fcbc137 | CAGAAATGTGTCGTGTGYCAGCMGCCGCGGTAA | 806rcbc237 | GGTTTAACACGCCCGGACTACNVGGGTWTCTAAT |
| D7 | 515fcbc8 | ATCCTTTGGTTCGTGTGYCAGCMGCCGCGGTAA | 806rcbc138 | GTAGAGGTAGAGCCGGACTACNVGGGTWTCTAAT |
| D8 | 515fcbc156 | TCGTGCGTGTTGGTGTGYCAGCMGCCGCGGTAA | 806rcbc11 | AATTGTGTCGGACCGGACTACNVGGGTWTCTAAT |
| D9 | 515fcbc10 | ACCGGTATGTACGTGTGYCAGCMGCCGCGGTAA | 806rcbc70 | GATTCCGGCTCACCGGACTACNVGGGTWTCTAAT |
| D10 | 515fcbc45 | TCGGAATTAGACGTGTGYCAGCMGCCGCGGTAA | 806rcbc5 | ATCGCACAGTAACCGGACTACNVGGGTWTCTAAT |
| D11 | 515fcbc96 | TACCGCTTCTTCGTGTGYCAGCMGCCGCGGTAA | 806rcbc185 | GGTTCGGTCCATCCGGACTACNVGGGTWTCTAAT |
| D12 | 515fcbc55 | TGTCGCAAATAGGTGTGYCAGCMGCCGCGGTAA | 806rcbc203 | CGGATAACCTCCCCGGACTACNVGGGTWTCTAAT |
| E1 | 515fcbc131 | ATCAGTACTAGGGTGTGYCAGCMGCCGCGGTAA | 806rcbc211 | TGCACGTGATAACCGGACTACNVGGGTWTCTAAT |
| E2 | 515fcbc7 | AGCGGAGGTTAGGTGTGYCAGCMGCCGCGGTAA | 806rcbc252 | CTATCATCCTCACCGGACTACNVGGGTWTCTAAT |
| E3 | 515fcbc89 | CTGCTATTCCTCGTGTGYCAGCMGCCGCGGTAA | 806rcbc197 | ATTCGGTAGTGCCCGGACTACNVGGGTWTCTAAT |
| E4 | 515fcbc134 | TGCAGCAAGATTGTGTGYCAGCMGCCGCGGTAA | 806rcbc206 | GCCTGTCTGCAACCGGACTACNVGGGTWTCTAAT |
| E5 | 515fcbc85 | GCGTTCTAGCTGGTGTGYCAGCMGCCGCGGTAA | 806rcbc263 | GATGCTGCCGTTCCGGACTACNVGGGTWTCTAAT |
| E6 | 515fcbc39 | TCGACATCTCTTGTGTGYCAGCMGCCGCGGTAA | 806rcbc41 | GAGCCATCTGTACCGGACTACNVGGGTWTCTAAT |
| E7 | 515fcbc11 | AATTGTGTCGGAGTGTGYCAGCMGCCGCGGTAA | 806rcbc248 | ACCGTGCTCACACCGGACTACNVGGGTWTCTAAT |
| E8 | 515fcbc25 | CGAGCAATCCTAGTGTGYCAGCMGCCGCGGTAA | 806rcbc235 | AGGTGAGTTCTACCGGACTACNVGGGTWTCTAAT |
| E9 | 515fcbc50 | GATGTTCGCTAGGTGTGYCAGCMGCCGCGGTAA | 806rcbc79 | GTGGTGGTTTCCCCGGACTACNVGGGTWTCTAAT |
| E10 | 515fcbc49 | GGCCAGTTCCTAGTGTGYCAGCMGCCGCGGTAA | 806rcbc78 | GTACGATATGACCCGGACTACNVGGGTWTCTAAT |
| E11 | 515fcbc20 | GATCTGCGATCCGTGTGYCAGCMGCCGCGGTAA | 806rcbc65 | ACTTCCAACTTCCCGGACTACNVGGGTWTCTAAT |
| E12 | 515fcbc13 | AGTCGAACGAGGGTGTGYCAGCMGCCGCGGTAA | 806rcbc54 | GTCGACAGAGGACCGGACTACNVGGGTWTCTAAT |
| F1 | 515fcbc130 | GCCAACAACCATGTGTGYCAGCMGCCGCGGTAA | 806rcbc258 | GAGAGTCCACTTCCGGACTACNVGGGTWTCTAAT |
| F2 | 515fcbc107 | TCACCTCCTTGTGTGTGYCAGCMGCCGCGGTAA | 806rcbc251 | CCTTGACCGATGCCGGACTACNVGGGTWTCTAAT |
| F3 | 515fcbc78 | GTACGATATGACGTGTGYCAGCMGCCGCGGTAA | 806rcbc157 | GTTATCGCATGGCCGGACTACNVGGGTWTCTAAT |
| F4 | 515fcbc120 | AGTAGCGGAAGAGTGTGYCAGCMGCCGCGGTAA | 806rcbc12 | TGCATACACTGGCCGGACTACNVGGGTWTCTAAT |
| F5 | 515fcbc35 | TACGAGCCCTAAGTGTGYCAGCMGCCGCGGTAA | 806rcbc141 | GGATCGTAATACCCGGACTACNVGGGTWTCTAAT |
| F6 | 515fcbc139 | CGTGATCCGCTAGTGTGYCAGCMGCCGCGGTAA | 806rcbc69 | TATCGACACAAGCCGGACTACNVGGGTWTCTAAT |
| F7 | 515fcbc102 | GTCACGGACATTGTGTGYCAGCMGCCGCGGTAA | 806rcbc261 | GGTCTCCTACAGCCGGACTACNVGGGTWTCTAAT |
| F8 | 515fcbc105 | ACTTGGTGTAAGGTGTGYCAGCMGCCGCGGTAA | 806rcbc145 | TGAACCCTATGGCCGGACTACNVGGGTWTCTAAT |
| F9 | 515fcbc40 | GAACACTTTGGAGTGTGYCAGCMGCCGCGGTAA | 806rcbc239 | GCCACGACTTACCCGGACTACNVGGGTWTCTAAT |
| F10 | 515fcbc48 | TACTACGTGGCCGTGTGYCAGCMGCCGCGGTAA | 806rcbc18 | CATTATGGCGTGCCGGACTACNVGGGTWTCTAAT |
| F11 | 515fcbc82 | ATGGCTGTCAGTGTGTGYCAGCMGCCGCGGTAA | 806rcbc44 | TAATACGGATCGCCGGACTACNVGGGTWTCTAAT |
| F12 | 515fcbc16 | GTAGATCGTGTAGTGTGYCAGCMGCCGCGGTAA | 806rcbc96 | TACCGCTTCTTCCCGGACTACNVGGGTWTCTAAT |
| G1 | 515fcbc27 | GTATCTGCGCGTGTGTGYCAGCMGCCGCGGTAA | 806rcbc22 | CAAACAACAGCTCCGGACTACNVGGGTWTCTAAT |
| G2 | 515fcbc136 | GATGTGGTGTTAGTGTGYCAGCMGCCGCGGTAA | 806rcbc61 | GTGGAGTCTCATCCGGACTACNVGGGTWTCTAAT |
| G3 | 515fcbc81 | TGCGCTGAATGTGTGTGYCAGCMGCCGCGGTAA | 806rcbc142 | GCATAGCATCAACCGGACTACNVGGGTWTCTAAT |
| G4 | 515fcbc65 | ACTTCCAACTTCGTGTGYCAGCMGCCGCGGTAA | 806rcbc207 | ACTGATGGCCTCCCGGACTACNVGGGTWTCTAAT |
| G5 | 515fcbc4 | TGGTCAACGATAGTGTGYCAGCMGCCGCGGTAA | 806rcbc257 | CCAGATATAGCACCGGACTACNVGGGTWTCTAAT |
| G6 | 515fcbc1 | ACGAGACTGATTGTGTGYCAGCMGCCGCGGTAA | 806rcbc205 | GACTTCATGCGACCGGACTACNVGGGTWTCTAAT |
| G7 | 515fcbc72 | GGTGACTAGTTCGTGTGYCAGCMGCCGCGGTAA | 806rcbc256 | CTTAGGCATGTGCCGGACTACNVGGGTWTCTAAT |
| G8 | 515fcbc70 | GATTCCGGCTCAGTGTGYCAGCMGCCGCGGTAA | 806rcbc67 | GTGTTGTCGTGCCCGGACTACNVGGGTWTCTAAT |
| G9 | 515fcbc87 | GGACTTCCAGCTGTGTGYCAGCMGCCGCGGTAA | 806rcbc30 | AGATTGACCAACCCGGACTACNVGGGTWTCTAAT |
| G10 | 515fcbc116 | CACTCATCATTCGTGTGYCAGCMGCCGCGGTAA | 806rcbc245 | TATCACCGGCACCCGGACTACNVGGGTWTCTAAT |
| G11 | 515fcbc157 | GTTATCGCATGGGTGTGYCAGCMGCCGCGGTAA | 806rcbc231 | GTGTTCCCAGAACCGGACTACNVGGGTWTCTAAT |
| G12 | 515fcbc84 | CGTAAGATGCCTGTGTGYCAGCMGCCGCGGTAA | 806rcbc201 | CGACTCTAAACGCCGGACTACNVGGGTWTCTAAT |
| H1 | 515fcbc155 | TCAGTTCTCGTTGTGTGYCAGCMGCCGCGGTAA | 806rcbc241 | GCCGTAAACTTGCCGGACTACNVGGGTWTCTAAT |
| H2 | 515fcbc143 | GTGTTAGATGTGGTGTGYCAGCMGCCGCGGTAA | 806rcbc25 | CGAGCAATCCTACCGGACTACNVGGGTWTCTAAT |
| H3 | 515fcbc51 | CTATCTCCTGTCGTGTGYCAGCMGCCGCGGTAA | 806rcbc101 | ACTCCTTGTGTTCCGGACTACNVGGGTWTCTAAT |
| H4 | 515fcbc149 | ACGACTGCATAAGTGTGYCAGCMGCCGCGGTAA | 806rcbc266 | TGGCTTTCTATCCCGGACTACNVGGGTWTCTAAT |
| H5 | 515fcbc18 | CATTATGGCGTGGTGTGYCAGCMGCCGCGGTAA | 806rcbc268 | GAGCGTATCCATCCGGACTACNVGGGTWTCTAAT |
| H6 | 515fcbc93 | TGGAGTAGGTGGGTGTGYCAGCMGCCGCGGTAA | 806rcbc151 | AGCTATGTATGGCCGGACTACNVGGGTWTCTAAT |
| H7 | 515fcbc144 | TTAGAGCCATGCGTGTGYCAGCMGCCGCGGTAA | 806rcbc260 | ACGTGTAGGCTTCCGGACTACNVGGGTWTCTAAT |
| H8 | 515fcbc133 | ACCCAAGCGTTAGTGTGYCAGCMGCCGCGGTAA | 806rcbc154 | CGTACTCTCGAGCCGGACTACNVGGGTWTCTAAT |
| H9 | 515fcbc101 | ACTCCTTGTGTTGTGTGYCAGCMGCCGCGGTAA | 806rcbc85 | GCGTTCTAGCTGCCGGACTACNVGGGTWTCTAAT |
| H10 | 515fcbc29 | CAAATTCGGGATGTGTGYCAGCMGCCGCGGTAA | 806rcbc23 | GCAACACCATCCCCGGACTACNVGGGTWTCTAAT |
| H11 | 515fcbc83 | GTTCTCTTCTCGGTGTGYCAGCMGCCGCGGTAA | 806rcbc149 | ACGACTGCATAACCGGACTACNVGGGTWTCTAAT |
| H12 | 515fcbc115 | TACCGAAGGTATGTGTGYCAGCMGCCGCGGTAA | 806rcbc130 | GCCAACAACCATCCGGACTACNVGGGTWTCTAAT |

Supplementary table 3 Alpha diversity statistics of fish skin and tank water microbiomes between different time points. Linear mixed effects model with random effects of the difference between differences compared to the control tank.

| Alpha diversity metric | Sample type | Timepoint 1 | Timepoint 2 | Treatment group | Estimate | Std. Error | t value | B.H. adjusted p value | Significance |
| --- | --- | --- | --- | --- | --- | --- | --- | --- | --- |
| Chao1 | Water | -2 | 7 | High | -17.36 | 141.28 | -0.13 | 1 | N.S. |
| Chao1 | Water | -2 | 7 | Low | -31.34 | 142.17 | -0.23 | 1 | N.S. |
| PD | Water | -2 | 7 | High | -1.67 | 15.27 | -0.12 | 1 | N.S. |
| PD | Water | -2 | 7 | Low | -3.98 | 15.55 | -0.28 | 1 | N.S. |
| Shannon | Water | -2 | 7 | High | -0.03 | 0.39 | -0.07 | 1 | N.S. |
| Shannon | Water | -2 | 7 | Low | -0.07 | 0.39 | -0.19 | 1 | N.S. |
| Chao1 | Water | 14 | 21 | High | 29.66 | 95.42 | 0.32 | 1 | N.S. |
| Chao1 | Water | 14 | 21 | Low | 39.34 | 96.72 | 0.42 | 1 | N.S. |
| PD | Water | 14 | 21 | High | 3.92 | 8.42 | 0.48 | 1 | N.S. |
| PD | Water | 14 | 21 | Low | 3.68 | 8.46 | 0.44 | 1 | N.S. |
| Shannon | Water | 14 | 21 | High | 0 | 0.5 | 0 | 1 | N.S. |
| Shannon | Water | 14 | 21 | Low | 0.13 | 0.46 | 0.29 | 1 | N.S. |
| Chao1 | Water | 21 | 28 | High | 43.75 | 92.2 | 0.49 | 1 | N.S. |
| Chao1 | Water | 21 | 28 | Low | 12.3 | 93.11 | 0.13 | 1 | N.S. |
| PD | Water | 21 | 28 | High | 4.73 | 8.44 | 0.57 | 1 | N.S. |
| PD | Water | 21 | 28 | Low | 0.52 | 8.59 | 0.06 | 1 | N.S. |
| Shannon | Water | 21 | 28 | High | 0.22 | 0.47 | 0.49 | 1 | N.S. |
| Shannon | Water | 21 | 28 | Low | 0.01 | 0.47 | 0.02 | 1 | N.S. |
| Chao1 | Water | 7 | 14 | High | 7.7 | 90.6 | 0.09 | 1 | N.S. |
| Chao1 | Water | 7 | 14 | Low | 18.82 | 90.77 | 0.22 | 1 | N.S. |
| PD | Water | 7 | 14 | High | 1.01 | 7.89 | 0.13 | 1 | N.S. |
| PD | Water | 7 | 14 | Low | 1.46 | 7.87 | 0.19 | 1 | N.S. |
| Shannon | Water | 7 | 14 | High | -0.17 | 0.46 | -0.39 | 1 | N.S. |
| Shannon | Water | 7 | 14 | Low | -0.04 | 0.45 | -0.08 | 1 | N.S. |
| Chao1 | Swab | -2 | 7 | High | 2.68 | 68.63 | 0.04 | 1 | N.S. |
| Chao1 | Swab | -2 | 7 | Low | -19.07 | 69.64 | -0.28 | 1 | N.S. |
| PD | Swab | -2 | 7 | High | -0.59 | 7.67 | -0.08 | 1 | N.S. |
| PD | Swab | -2 | 7 | Low | -2.59 | 7.59 | -0.35 | 1 | N.S. |
| Shannon | Swab | -2 | 7 | High | 0.01 | 0.57 | 0.01 | 1 | N.S. |
| Shannon | Swab | -2 | 7 | Low | 0.04 | 0.58 | 0.07 | 1 | N.S. |
| Chao1 | Swab | 14 | 21 | High | 10.54 | 58.51 | 0.19 | 1 | N.S. |
| Chao1 | Swab | 14 | 21 | Low | 24.48 | 57.8 | 0.43 | 1 | N.S. |
| PD | Swab | 14 | 21 | High | 1.35 | 5.28 | 0.26 | 1 | N.S. |
| PD | Swab | 14 | 21 | Low | 2.18 | 5.22 | 0.43 | 1 | N.S. |
| Shannon | Swab | 14 | 21 | High | 0.01 | 0.37 | 0.03 | 1 | N.S. |
| Shannon | Swab | 14 | 21 | Low | 0.3 | 0.34 | 0.92 | 1 | N.S. |
| Chao1 | Swab | 21 | 28 | High | 19.49 | 58.43 | 0.34 | 1 | N.S. |
| Chao1 | Swab | 21 | 28 | Low | 33.32 | 58.48 | 0.59 | 1 | N.S. |
| PD | Swab | 21 | 28 | High | 1.83 | 5.14 | 0.37 | 1 | N.S. |
| PD | Swab | 21 | 28 | Low | 3.1 | 5.17 | 0.62 | 1 | N.S. |
| Shannon | Swab | 21 | 28 | High | 0.17 | 0.35 | 0.49 | 1 | N.S. |
| Shannon | Swab | 21 | 28 | Low | 0.33 | 0.35 | 0.95 | 1 | N.S. |
| Chao1 | Swab | 7 | 14 | High | 3.35 | 58.5 | 0.06 | 1 | N.S. |
| Chao1 | Swab | 7 | 14 | Low | 5.78 | 59.26 | 0.1 | 1 | N.S. |
| PD | Swab | 7 | 14 | High | 0.34 | 4.93 | 0.07 | 1 | N.S. |
| PD | Swab | 7 | 14 | Low | 0.27 | 4.93 | 0.06 | 1 | N.S. |
| Shannon | Swab | 7 | 14 | High | -0.06 | 0.44 | -0.14 | 1 | N.S. |
| Shannon | Swab | 7 | 14 | Low | 0.11 | 0.44 | 0.27 | 1 | N.S. |

Supplementary Table 4 Pairwise adonis2 (PERMANOVA) statistics table on a Bray-Curtis dissimilarity matrix showing the difference between carp skin swab treatment groups at different time points. **p < 0.01, *p < 0.05, N.S. not significant (p > 0.05).

| Treatment 1 | Treatment 2 | Timepoint | Pairwise adonis B.H. adjusted p-value | Pairwise adonis R^2^ (%) |
| --- | --- | --- | --- | --- |
| **High** | Control | -2 | 0.08 | 3.87; N.S |
| **High** | Low | -2 | 0.03 | 5.86 * |
| **Low** | Control | -2 | 0.03 | 5.94 * |
| **High** | Control | 7 | p < 0.01 | 27.68 ** |
| **High** | Low | 7 | p < 0.01 | 28.35 ** |
| **Low** | Control | 7 | p < 0.01 | 26.76 ** |
| **High** | Control | 14 | p < 0.01 | 33.39 ** |
| **High** | Low | 14 | p < 0.01 | 35.09 ** |
| **Low** | Control | 14 | p < 0.01 | 24.06 ** |
| **High** | Control | 21 | p < 0.01 | 12.97 ** |
| **High** | Low | 21 | p < 0.01 | 13.73 ** |
| **Low** | Control | 21 | p < 0.01 | 12.19 ** |
| **High** | Control | 28 | p < 0.01 | 20.15 ** |
| **High** | Low | 28 | p < 0.01 | c ** |
| **Low** | Control | 28 | p < 0.01 | 18.27 ** |

Supplementary Table 5 Pairwise adonis2 (PERMANOVA) statistics table on a Bray-Curtis dissimilarity matrix showing the difference between tank water (filter) treatment groups at different time points. **p < 0.01, N.S. not significant (p > 0.05).

| Treatment 1 | Treatment 2 | Timepoint | Pairwise adonis B.H. adjusted p-value | Pairwise adonis R^2^ (%) |
| --- | --- | --- | --- | --- |
| **High** | Control | -2 | 0.12 | 13.01; N.S. |
| **High** | Low | -2 | 0.2 | 11.94; N.S. |
| **Low** | Control | -2 | 0.07 | 20.83; N.S. |
| **High** | Control | 7 | 0.1 | 19.59; N.S. |
| **High** | Low | 7 | 0.01 | 40.59 ** |
| **Low** | Control | 7 | 0.01 | 40.79 ** |
| **High** | Control | 14 | 0.01 | 39.66 ** |
| **High** | Low | 14 | 0.01 | 48.47 ** |
| **Low** | Control | 14 | 0.01 | 42.47 ** |
| **High** | Control | 21 | 0.11 | 15.83; N.S. |
| **High** | Low | 21 | 0.06 | 19.23; N.S. |
| **Low** | Control | 21 | 0.23 | 12.22; N.S. |
| **High** | Control | 28 | 0.08 | 17.3; N.S. |
| **High** | Low | 28 | 0.08 | 20.91; N.S. |
| **Low** | Control | 28 | 0.15 | 14.83; N.S. |

Supplementary Table 6 Beta diversity statistics were tested using a pairwise.adonis2 (PERMANOVA) of Aitchison’s Distance, Binary Jaccard, Bray-Curtis, Weighted UniFrac and Weighted UniFrac 0.5 (generalised UniFrac with 0.5 taxonomic weighting).

| Treatment 1 | Treatment 2 | Sample type | Timepoint | Pairwise adonis B.H. adjusted p value | Pairwise adonis R^2^ percentage | Significance | Distance matrix |
| --- | --- | --- | --- | --- | --- | --- | --- |
| High | Control | Water | -2 | 0.11 | 11.05 | N.S. | Aitchison’s Distance |
| High | Control | Water | -2 | 0.25 | 9.81 | N.S. | Binary Jaccard |
| High | Control | Water | -2 | 0.12 | 13.01 | N.S. | Bray-Curtis |
| High | Control | Water | -2 | 0.11 | 19.67 | N.S. | Weighted UniFrac |
| High | Control | Water | -2 | 0.20 | 13.79 | N.S. | Weighted UniFrac 0.5 |
| High | Low | Water | -2 | 0.08 | 11.98 | N.S. | Aitchison’s Distance |
| High | Low | Water | -2 | 0.19 | 10.26 | N.S. | Binary Jaccard |
| High | Low | Water | -2 | 0.20 | 11.94 | N.S. | Bray-Curtis |
| High | Low | Water | -2 | 0.32 | 10.67 | N.S. | Weighted UniFrac |
| High | Low | Water | -2 | 0.27 | 11.01 | N.S. | Weighted UniFrac 0.5 |
| Low | Control | Water | -2 | 0.08 | 12.16 | N.S. | Aitchison’s Distance |
| Low | Control | Water | -2 | 0.12 | 11.13 | N.S. | Binary Jaccard |
| Low | Control | Water | -2 | 0.07 | 20.83 | N.S. | Bray-Curtis |
| Low | Control | Water | -2 | 0.06 | 29.75 | N.S. | Weighted UniFrac |
| Low | Control | Water | -2 | 0.11 | 20.54 | N.S. | Weighted UniFrac 0.5 |
| High | Control | Water | 7 | p < 0.01 | 17.64 | ** | Aitchison’s Distance |
| High | Control | Water | 7 | p < 0.01 | 16.53 | ** | Binary Jaccard |
| High | Control | Water | 7 | 0.10 | 19.59 | N.S. | Bray-Curtis |
| High | Control | Water | 7 | 0.66 | 3.78 | N.S. | Weighted UniFrac |
| High | Control | Water | 7 | 0.55 | 6.22 | N.S. | Weighted UniFrac 0.5 |
| High | Low | Water | 7 | p < 0.01 | 17.62 | ** | Aitchison’s Distance |
| High | Low | Water | 7 | 0.01 | 14.76 | ** | Binary Jaccard |
| High | Low | Water | 7 | 0.01 | 40.59 | ** | Bray-Curtis |
| High | Low | Water | 7 | 0.01 | 48.49 | * | Weighted UniFrac |
| High | Low | Water | 7 | 0.01 | 47.08 | * | Weighted UniFrac 0.5 |
| Low | Control | Water | 7 | p < 0.01 | 22.00 | ** | Aitchison’s Distance |
| Low | Control | Water | 7 | p < 0.01 | 16.66 | ** | Binary Jaccard |
| Low | Control | Water | 7 | 0.01 | 40.79 | ** | Bray-Curtis |
| Low | Control | Water | 7 | 0.01 | 56.11 | ** | Weighted UniFrac |
| Low | Control | Water | 7 | 0.01 | 56.14 | ** | Weighted UniFrac 0.5 |
| High | Control | Water | 14 | p < 0.01 | 16.20 | ** | Aitchison’s Distance |
| High | Control | Water | 14 | p < 0.01 | 16.72 | ** | Binary Jaccard |
| High | Control | Water | 14 | 0.01 | 39.66 | ** | Bray-Curtis |
| High | Control | Water | 14 | 0.02 | 39.63 | * | Weighted UniFrac |
| High | Control | Water | 14 | 0.05 | 23.43 | * | Weighted UniFrac 0.5 |
| High | Low | Water | 14 | p < 0.01 | 18.12 | ** | Aitchison’s Distance |
| High | Low | Water | 14 | p < 0.01 | 16.12 | ** | Binary Jaccard |
| High | Low | Water | 14 | 0.01 | 48.47 | ** | Bray-Curtis |
| High | Low | Water | 14 | 0.01 | 36.35 | * | Weighted UniFrac |
| High | Low | Water | 14 | 0.11 | 18.59 | N.S. | Weighted UniFrac 0.5 |
| Low | Control | Water | 14 | p < 0.01 | 19.73 | ** | Aitchison’s Distance |
| Low | Control | Water | 14 | p < 0.01 | 15.79 | ** | Binary Jaccard |
| Low | Control | Water | 14 | 0.01 | 42.47 | ** | Bray-Curtis |
| Low | Control | Water | 14 | 0.04 | 25.99 | * | Weighted UniFrac |
| Low | Control | Water | 14 | 0.04 | 26.27 | * | Weighted UniFrac 0.5 |
| High | Control | Water | 21 | p < 0.01 | 15.94 | ** | Aitchison’s Distance |
| High | Control | Water | 21 | p < 0.01 | 18.55 | ** | Binary Jaccard |
| High | Control | Water | 21 | 0.11 | 15.83 | N.S. | Bray-Curtis |
| High | Control | Water | 21 | 0.21 | 16.04 | N.S. | Weighted UniFrac |
| High | Control | Water | 21 | 0.12 | 17.93 | N.S. | Weighted UniFrac 0.5 |
| High | Low | Water | 21 | 0.01 | 16.04 | ** | Aitchison’s Distance |
| High | Low | Water | 21 | p < 0.01 | 15.45 | ** | Binary Jaccard |
| High | Low | Water | 21 | 0.06 | 19.23 | N.S. | Bray-Curtis |
| High | Low | Water | 21 | 0.04 | 33.72 | * | Weighted UniFrac |
| High | Low | Water | 21 | 0.02 | 30.35 | * | Weighted UniFrac 0.5 |
| Low | Control | Water | 21 | p < 0.01 | 17.36 | ** | Aitchison’s Distance |
| Low | Control | Water | 21 | p < 0.01 | 14.86 | ** | Binary Jaccard |
| Low | Control | Water | 21 | 0.23 | 12.22 | N.S. | Bray-Curtis |
| Low | Control | Water | 21 | 0.33 | 10.45 | N.S. | Weighted UniFrac |
| Low | Control | Water | 21 | 0.24 | 12.41 | N.S. | Weighted UniFrac 0.5 |
| High | Control | Water | 28 | p < 0.01 | 16.83 | ** | Aitchison’s Distance |
| High | Control | Water | 28 | p < 0.01 | 18.84 | ** | Binary Jaccard |
| High | Control | Water | 28 | 0.08 | 17.30 | N.S. | Bray-Curtis |
| High | Control | Water | 28 | 0.40 | 9.07 | N.S. | Weighted UniFrac |
| High | Control | Water | 28 | 0.22 | 12.91 | N.S. | Weighted UniFrac 0.5 |
| High | Low | Water | 28 | p < 0.01 | 19.09 | ** | Aitchison’s Distance |
| High | Low | Water | 28 | p < 0.01 | 16.14 | ** | Binary Jaccard |
| High | Low | Water | 28 | 0.08 | 20.91 | N.S. | Bray-Curtis |
| High | Low | Water | 28 | 0.02 | 38.39 | * | Weighted UniFrac |
| High | Low | Water | 28 | 0.02 | 33.98 | * | Weighted UniFrac 0.5 |
| Low | Control | Water | 28 | 0.02 | 13.65 | * | Aitchison’s Distance |
| Low | Control | Water | 28 | 0.01 | 13.55 | * | Binary Jaccard |
| Low | Control | Water | 28 | 0.15 | 14.83 | N.S. | Bray-Curtis |
| Low | Control | Water | 28 | 0.25 | 13.38 | N.S. | Weighted UniFrac |
| Low | Control | Water | 28 | 0.21 | 14.38 | N.S. | Weighted UniFrac 0.5 |
| High | Control | Swab | -2 | p < 0.01 | 3.76 | ** | Aitchison’s Distance |
| High | Control | Swab | -2 | p < 0.01 | 3.48 | ** | Binary Jaccard |
| High | Control | Swab | -2 | 0.08 | 3.87 | N.S. | Bray-Curtis |
| High | Control | Swab | -2 | 0.13 | 4.36 | N.S. | Weighted UniFrac |
| High | Control | Swab | -2 | 0.18 | 3.43 | N.S. | Weighted UniFrac 0.5 |
| High | Low | Swab | -2 | p < 0.01 | 4.77 | ** | Aitchison’s Distance |
| High | Low | Swab | -2 | p < 0.01 | 3.68 | ** | Binary Jaccard |
| High | Low | Swab | -2 | 0.03 | 5.86 | * | Bray-Curtis |
| High | Low | Swab | -2 | 0.22 | 3.37 | N.S. | Weighted UniFrac |
| High | Low | Swab | -2 | 0.22 | 3.08 | N.S. | Weighted UniFrac 0.5 |
| Low | Control | Swab | -2 | p < 0.01 | 4.08 | ** | Aitchison’s Distance |
| Low | Control | Swab | -2 | p < 0.01 | 3.66 | ** | Binary Jaccard |
| Low | Control | Swab | -2 | 0.03 | 5.94 | * | Bray-Curtis |
| Low | Control | Swab | -2 | 0.21 | 3.63 | N.S. | Weighted UniFrac |
| Low | Control | Swab | -2 | 0.22 | 3.07 | N.S. | Weighted UniFrac 0.5 |
| High | Control | Swab | 7 | p < 0.01 | 10.37 | ** | Aitchison’s Distance |
| High | Control | Swab | 7 | p < 0.01 | 8.99 | ** | Binary Jaccard |
| High | Control | Swab | 7 | p < 0.01 | 27.68 | ** | Bray-Curtis |
| High | Control | Swab | 7 | p < 0.01 | 14.78 | ** | Weighted UniFrac |
| High | Control | Swab | 7 | 0.01 | 12.29 | ** | Weighted UniFrac 0.5 |
| High | Low | Swab | 7 | p < 0.01 | 9.97 | ** | Aitchison’s Distance |
| High | Low | Swab | 7 | p < 0.01 | 8.13 | ** | Binary Jaccard |
| High | Low | Swab | 7 | p < 0.01 | 28.35 | ** | Bray-Curtis |
| High | Low | Swab | 7 | p < 0.01 | 31.69 | bdisp_sig | Weighted UniFrac |
| High | Low | Swab | 7 | p < 0.01 | 30.61 | ** | Weighted UniFrac 0.5 |
| Low | Control | Swab | 7 | p < 0.01 | 12.65 | ** | Aitchison’s Distance |
| Low | Control | Swab | 7 | p < 0.01 | 9.64 | ** | Binary Jaccard |
| Low | Control | Swab | 7 | p < 0.01 | 26.76 | ** | Bray-Curtis |
| Low | Control | Swab | 7 | p < 0.01 | 50.01 | ** | Weighted UniFrac |
| Low | Control | Swab | 7 | p < 0.01 | 46.33 | ** | Weighted UniFrac 0.5 |
| High | Control | Swab | 14 | p < 0.01 | 10.10 | ** | Aitchison’s Distance |
| High | Control | Swab | 14 | p < 0.01 | 9.37 | ** | Binary Jaccard |
| High | Control | Swab | 14 | p < 0.01 | 33.39 | ** | Bray-Curtis |
| High | Control | Swab | 14 | p < 0.01 | 32.96 | ** | Weighted UniFrac |
| High | Control | Swab | 14 | p < 0.01 | 22.18 | ** | Weighted UniFrac 0.5 |
| High | Low | Swab | 14 | p < 0.01 | 9.66 | bdisp_sig | Aitchison’s Distance |
| High | Low | Swab | 14 | p < 0.01 | 7.33 | ** | Binary Jaccard |
| High | Low | Swab | 14 | p < 0.01 | 35.09 | ** | Bray-Curtis |
| High | Low | Swab | 14 | p < 0.01 | 20.93 | ** | Weighted UniFrac |
| High | Low | Swab | 14 | p < 0.01 | 13.36 | ** | Weighted UniFrac 0.5 |
| Low | Control | Swab | 14 | p < 0.01 | 11.99 | bdisp_sig | Aitchison’s Distance |
| Low | Control | Swab | 14 | p < 0.01 | 10.52 | ** | Binary Jaccard |
| Low | Control | Swab | 14 | p < 0.01 | 24.06 | ** | Bray-Curtis |
| Low | Control | Swab | 14 | p < 0.01 | 11.70 | ** | Weighted UniFrac |
| Low | Control | Swab | 14 | p < 0.01 | 13.96 | ** | Weighted UniFrac 0.5 |
| High | Control | Swab | 21 | p < 0.01 | 7.49 | ** | Aitchison’s Distance |
| High | Control | Swab | 21 | p < 0.01 | 8.89 | bdisp_sig | Binary Jaccard |
| High | Control | Swab | 21 | p < 0.01 | 12.97 | ** | Bray-Curtis |
| High | Control | Swab | 21 | 0.01 | 9.07 | * | Weighted UniFrac |
| High | Control | Swab | 21 | 0.05 | 5.87 | * | Weighted UniFrac 0.5 |
| High | Low | Swab | 21 | p < 0.01 | 6.61 | ** | Aitchison’s Distance |
| High | Low | Swab | 21 | p < 0.01 | 7.57 | ** | Binary Jaccard |
| High | Low | Swab | 21 | p < 0.01 | 13.73 | ** | Bray-Curtis |
| High | Low | Swab | 21 | 0.06 | 6.23 | N.S. | Weighted UniFrac |
| High | Low | Swab | 21 | 0.01 | 9.45 | ** | Weighted UniFrac 0.5 |
| Low | Control | Swab | 21 | p < 0.01 | 8.81 | bdisp_sig | Aitchison’s Distance |
| Low | Control | Swab | 21 | p < 0.01 | 8.20 | ** | Binary Jaccard |
| Low | Control | Swab | 21 | p < 0.01 | 12.19 | ** | Bray-Curtis |
| Low | Control | Swab | 21 | p < 0.01 | 12.08 | ** | Weighted UniFrac |
| Low | Control | Swab | 21 | p < 0.01 | 11.32 | ** | Weighted UniFrac 0.5 |
| High | Control | Swab | 28 | p < 0.01 | 8.77 | ** | Aitchison’s Distance |
| High | Control | Swab | 28 | p < 0.01 | 10.13 | ** | Binary Jaccard |
| High | Control | Swab | 28 | p < 0.01 | 20.15 | ** | Bray-Curtis |
| High | Control | Swab | 28 | p < 0.01 | 17.33 | ** | Weighted UniFrac |
| High | Control | Swab | 28 | p < 0.01 | 16.56 | ** | Weighted UniFrac 0.5 |
| High | Low | Swab | 28 | p < 0.01 | 8.75 | ** | Aitchison’s Distance |
| High | Low | Swab | 28 | p < 0.01 | 7.99 | ** | Binary Jaccard |
| High | Low | Swab | 28 | p < 0.01 | 16.33 | ** | Bray-Curtis |
| High | Low | Swab | 28 | p < 0.01 | 16.81 | ** | Weighted UniFrac |
| High | Low | Swab | 28 | p < 0.01 | 15.81 | ** | Weighted UniFrac 0.5 |
| Low | Control | Swab | 28 | p < 0.01 | 9.98 | bdisp_sig | Aitchison’s Distance |
| Low | Control | Swab | 28 | p < 0.01 | 9.10 | ** | Binary Jaccard |
| Low | Control | Swab | 28 | p < 0.01 | 18.27 | ** | Bray-Curtis |
| Low | Control | Swab | 28 | 0.01 | 10.26 | ** | Weighted UniFrac |
| Low | Control | Swab | 28 | 0.01 | 10.90 | bdisp_sig | Weighted UniFrac 0.5 |

Supplementary Table 7 Differential abundance of fish skin and tank water microbiomes of high and lose concentration exposure groups compared to the control. Statistics were tested using a linear mixed model with random effects.

Supplementary Table 7 submitted as a separate file due to large amounts of data. See: “diff.abund.taxa.csv/.xlsx”

# Supplementary Documents

## Supplementary Methods

## DNA extraction and 16S rRNA sequencing

All PCRs were performed in duplicate with a total reaction volume of 25 μL per reaction, consisting of 12.5 μL of Platinum SuperFi II Master Mix DNA Polymerase (ThermoFisher; 12368010), 9 μL of nuclease free water, 1.25 μL of forward and reverse primers each (0.5 μM final reaction concentration), and 1 μL of DNA extract. PCR conditions consisted of an initial denaturing step at 98°C for 30seconds, 30 cycles of denaturing at 98°C for 10 seconds, annealing at 61°C for 20seconds and extension at 72°C for 30 seconds, with a final extension step of 72°Cfor 2 minutes. Amplicon concentrations were determined using theQuantiFluor dsDNA System (E2670, Promega) in 96-well plate format. Pooled plates were gel purified of non-target DNA, residual primer dimers and othermolecular matter using a Qiagen MinElute Gel Extraction Kit (28604) as outlinedby the manufacturer’s instructions. Gel purified pools were confirmed to haveamplicons of expected size and concentration using 1 µL of pooled DNA on the AgilentD1000 ScreenTape System and 2 µL Qubit Broad Range dsDNA Quantification AssayKits (Invitrogen; Q32850) following the manufacturer's recommended protocol

## 16S Demultiplexing and bioinformatic processing

After sequencing, 16S amplicon sequences were demultiplexed using Illumina barcodes (to plate level) by the Exeter Sequencing Centre. The cutadapt algorithm v4.5 (Martin, 2011) was then used to demultiplexed to sample (well) level keeping only reads with both dual-indexes (Supplementary Table 1) and allowing for a maximum of two mismatches on dual indexes for sample identification. Any nucleotides with a quality score of Q2 or lower, containing “N”s as base pairs or were identified as PhiX sequences were removed. Forward reads were truncated at position 216 and reverse reads at 217 based upon manual inspection of read quality profiles in R via DADA2 v1.28.0 (Callahan et al., 2016). As a NovaSeq error correction model has not been implemented in DADA2, four different error rates were learnt using a minimum of 1 billion bases for both the forward and reverse reads (hhollandmoritz, 2021). The loessErrfun_mod4 which alters the loess function and enforces monotonicity manually, was selected as the best model for correcting both the forward and reverse reads based on nucleotide error frequency versus consensus quality scores. Unique reads were dereplicated and forward and reverse reads merged into amplicon sequence variants (ASVs) producing an ASV count table. Chimeric sequences were removed and only ASVs between 252 and 254 base pairs were retained. PCR-free ligation of Illumina adaptors resulted in reads occurring both in the forward and reverse complement orientation due to the non-specific binding of Illumina sequencing adaptors to either the forward or reverse orientation of reads. Approximately 50% of reads were in the reverse complement orientation and were orientated to the forward orientation before taxonomy was assigned using the SILVA database (Quast et al., 2013) based on the nr99_v138.1_train_set to genus level. A phylogenetic tree was constructed from ASVs using IQ-TREE v2.2.5 (Nguyen et al., 2015) algorithm with an extended model selection followed by tree inference.

## Shotgun metagenomic sequencing

Subsequent bioinformatic QC was performed by the Centre for Genomics Research using the following steps: The raw Fastq files are trimmed for the presence of Illumina adapter sequences using Cutadapt version 1.2.1 (Martin, 2011). The option -O 3 was used, so the 3' end of any reads which match the adapter sequence for 3 bp or more are trimmed. The reads are further trimmed using Sickle version 1.200 (Joshi & Fass, 2011) with a minimum window quality score of 20. Reads shorter than 15 bp. after trimming were removed.

## Antibiotic analysis of the water

At each antibiotic sampling timepoint, 100 mL of tank water was collected in duplicate and stored in 100 mL glass DURAN^®^ bottles with PTFE lids until filtration onto HLB cartridges later the same day. All samples were spiked with 100 ng of each internal standard reconstituted in methanol as soon as possible consisting of clarithromycin-N-methyl-d3 (Toronto Research Chemicals; TRC-C559752), desmethyl Ofloxacin-d8 (Toronto Research Chemicals; TRC-D292097), sulfamethoxazole-d4 (Toronto Research Chemicals; TRC-S699087) and trimethoprim-d9 (Major) (Toronto Research Chemicals; TRC-T795617).

Water samples were passed through Hydrophilic-Lipophilic-Balanced solid phase extraction cartridges (Waters; SKU: WAT094226), using a vacuum manifold at a flow rate of ~3 mL min^-1^ (roughly one drop per second). Marprene tubing rinsed in methanol then ultrapure water was used to load samples between the cartridge and sample bottles. The vacuum manifold was left to run 30 minutes after the sample had been filtered onto the cartridge to ensure the cartridges were dry. Dried cartridges were sealed in parafilm then aluminium foil and stored at -20°C to prevent (UV) degradation until analysis.

Cartridges were shipped on dry ice to the University of Bath for analysis. Samples were eluted from cartridges using 4 mL of methanol (MeOH, Sigma-Aldrich) into silanized glass vials (Thermo Scientific). They were then evaporated to dryness under a stream of nitrogen at 40°C using a TurboVap LV evaporator, reconstituted using 500 µL 80:20 H_2_O:MeOH and transferred to polypropylene LC vials (Waters, UK) for analysis. Quantification was performed using ultra high-performance liquid chromatography with tandem mass spectrometry (UPLC-MS/MS) using multiple reaction monitoring (MRM), based on a method validated by Holton & Kasprzyk-Hordern (2021). UPLC was performed using an ACQUITY UPLC^TM^ system (Waters, UK) prior to analysis by a Xevo triple quadrupole mass spectrometer with positive electrospray ionisation (ESI) (Xevo TQD, Waters, UK). MS settings were as follows: capillary voltage 3.20 kV, source temperature 150 °C, and desolvation temperature 400 °C. Cone gas flow was 100 Lh^-1^ and desolvation gas flow 600 Lh^-1^ (both nitrogen). The collision gas was argon. Data processing was performed using MassLynx and TargetLynx software (Waters Laboratory Informatics Software). Method limits of detection and quantification were ≤ 0.75 ng/L and ≤ 2.5 ng/L for all analytes measured in this study. Quality assurance was assessed by injection of mobile phase QCs and solvent blanks throughout the run, to assess instrument performance and to identify any contamination or carryover between samples. Mobile phase QCs comprising H_2_O/MeOH (80/20, v,v) were prepared at 10, 50, and 100 µg/L analyte concentration levels from 1 µg/mL mixtures of analyte standards and isotopically labelled internal standards of analytical grade (Sigma-Aldrich, Gillingham, UK, and TRC, Toronto, Canada). They were injected in triplicate at a minimum, to assess intra and inter-day precision of the instrument. Instrument accuracy and precision were as follows: sulfamethoxazole 93 ± 5%, trimethoprim 159 ± 30%, ciprofloxacin 141 ± 26%, tetracycline 83 ± 17%, and clarithromycin 55 ± 3%. For more information and method validation parameters, see Holton & Kasprzyk-Hordern (2021). All solvents used for sample reconstitution and analysis were HPLC grade, and water used in sample preparation was Mili-Q (ultrapure 18.2 MΩ/cm).

# References

Bengtsson-Palme, J. and Larsson, D.G.J. (2016) ‘Concentrations of antibiotics predicted to select for resistant bacteria: Proposed limits for environmental regulation’, *Environment International*, 86, pp. 140–149. Available at: https://doi.org/10.1016/j.envint.2015.10.015.

Booth, A., Aga, D.S. and Wester, A.L. (2020) ‘Retrospective analysis of the global antibiotic residues that exceed the predicted no effect concentration for antimicrobial resistance in various environmental matrices’, *Environment International*, 141, p. 105796. Available at: https://doi.org/10.1016/j.envint.2020.105796.

Danner, M.C. *et al.* (2019) ‘Antibiotic pollution in surface fresh waters: Occurrence and effects’, *Science of the Total Environment*. Elsevier, pp. 793–804. Available at: https://doi.org/10.1016/j.scitotenv.2019.01.406.

Holton, E. and Kasprzyk-Hordern, B. (2021) ‘Multiresidue antibiotic-metabolite quantification method using ultra-performance liquid chromatography coupled with tandem mass spectrometry for environmental and public exposure estimation’, *Analytical and Bioanalytical Chemistry*, 413(23). Available at: https://doi.org/10.1007/s00216-021-03573-4.

Sanseverino, I. *et al.* (2018) ‘State of the Art on the Contribution of Water to Antimicrobial Resistance’, *European Commission* [Preprint]. Available at: https://doi.org/10.2760/771124.

Wang, J. *et al.* (2020) ‘Occurrence and fate of antibiotics, antibiotic resistant genes (ARGs) and antibiotic resistant bacteria (ARB) in municipal wastewater treatment plant: An overview’, *Science of The Total Environment*, 744, p. 140997. Available at: https://doi.org/10.1016/j.scitotenv.2020.140997.
